# IκB kinase β (IKKβ): Structure, transduction mechanism, biological function, and discovery of its inhibitors

**DOI:** 10.7150/ijbs.85158

**Published:** 2023-08-06

**Authors:** Juan Zhang, Rui Zhang, Wei Li, Xiao-Chi Ma, Feng Qiu, Cheng-Peng Sun

**Affiliations:** 1 School of Chinese Materia Medica, State Key Laboratory of Component-Based Chinese Medicine, Tianjin State Key Laboratory of Modern Chinese Medicine, Tianjin University of Traditional Chinese Medicine, Tianjin 301617, China.; 2 College of Pharmacy, Second Affiliated Hospital, Dalian Medical University, Dalian 116044, China.; 3 School of Pharmaceutical Sciences, Health Science Center, Shenzhen University, Shenzhen 518061, China.; 4 Faculty of Pharmaceutical Sciences, Toho University, Chiba 274-8510, Japan.

**Keywords:** IKKβ, Inhibitor, Natural products, chemical synthesis, Bioactivity

## Abstract

The effective approach to discover innovative drugs will ask natural products for answers because of their complex and changeable structures and multiple biological activities. Inhibitory kappa B kinase beta (IKKβ), known as IKK2, is a key regulatory kinase responsible for the activation of NF-κB through its phosphorylation at Ser177 and Ser181 to promote the phosphorylation of inhibitors of kappa B (IκBs), triggering their ubiquitination and degradation to active the nuclear factor kappa-B (NF-κB) cascade. Chemical inhibition of IKKβ or its genetic knockout has become an effective method to block NF-κB-mediated proliferation and migration of tumor cells and inflammatory response. In this review, we summarized the structural feature and transduction mechanism of IKKβ and the discovery of inhibitors from natural resources (e.g. sesquiterpenoids, diterpenoids, triterpenoids, flavonoids, and alkaloids) and chemical synthesis (e.g. pyrimidines, pyridines, pyrazines, quinoxalines, thiophenes, and thiazolidines). In addition, the biosynthetic pathway of novel natural IKKβ inhibitors and their biological potentials were discussed. This review will provide inspiration for the structural modification of IKKβ inhibitors based on the skeleton of natural products or chemical synthesis and further phytochemistry investigations.

## Introduction

In 1986, Sen and co-workers focused on the work to discover novel nuclear factors that regulated the expression of immunoglobulin G (IgG) in B cells 1. Fortunately, they found that a nuclear factor specifically bound to the promoter region of the Ig κ light chain, namely nuclear factor kappa-B (NF-κB) 2. During this initial pioneering work and the subsequent recognition of transcription factors, NF-κB has been served as the most well-studied signal transduction paradigm in the latest three decades 2, 3.

NF-κB comprises five members of the family Rel, such as RelA (p65), RelB, c-Rel, NF-κB1 (p50), and NF-κB2 (p52) 4. In normal cells, they form the homo- or hetero-complex with inhibitor of kappa B (IκB, including IκBα, IκBβ, and IκBε) and are anchored in the cytoplasm 4. After the stimulation of exogenous substances, e.g. lipopolysaccharide (LPS), or inflammatory factors, e.g. interleukin-6 (IL-6) and tumor necrosis factor alpha (TNF-α), IκBα is phosphorylated at Ser32 and Ser36, resulting in the rapid ubiquitination by the proteosome in the dependent manner to release NF-κB 5, 6. Furthermore, activation of NF-κB leads to its nuclear translocation from the cytoplasm, allowing the binding of the promoter of its response elements and enhancer regions of its responsive genes, such as inflammatory cytokines, adhesion molecules, chemokines, and transcription factors 7. However, it is very difficult to identify which can promote the phosphorylation of IκBs, which is not achieved until the presence of inhibitory kappa B kinases (IKKs) 3. IKKβ, also known as IKK2, is a key regulatory kinase responsible for the NF-κB activation. In the classical pathway, the phosphorylation of IKKβ at Ser177 and Ser181, through the co-localization with TGF-beta-activated kinase 1 (TAK1) and mitogen-activated protein kinase kinase kinase 3 (MEKK3), promotes the phosphorylation of IκBs, triggering their ubiquitination and degradation to worsen the NF-κB cascade 3, 8, 9.

Because activated IKKβ can rapidly phosphorylate IκBs, chemical inhibition of IKKβ or its genetic knockout (KO) has become an experimental approach to block NF-κB mediated proliferation and migration of tumor cells and inflammatory response 3, 8, 9. For example, rectal carcinoma can be alleviated via suppressing the IKKβ activity. Furthermore, the phosphorylation level of IKKβ is upregulated in melanoma tumor, and its genetic deletion noteworthily suppresses the development of melanoma tumor 10. Herein, focused on the regulation and function of IKKβ, we summarized its inhibitors from natural products and chemical synthesis, which provided a useful guidance for the future development of potential IKKβ inhibitors, and the relationship between IKKβ and cancer, central nervous system (CNS), and metabolic diseases.

## Structure of IKKβ

**Figure 1:**
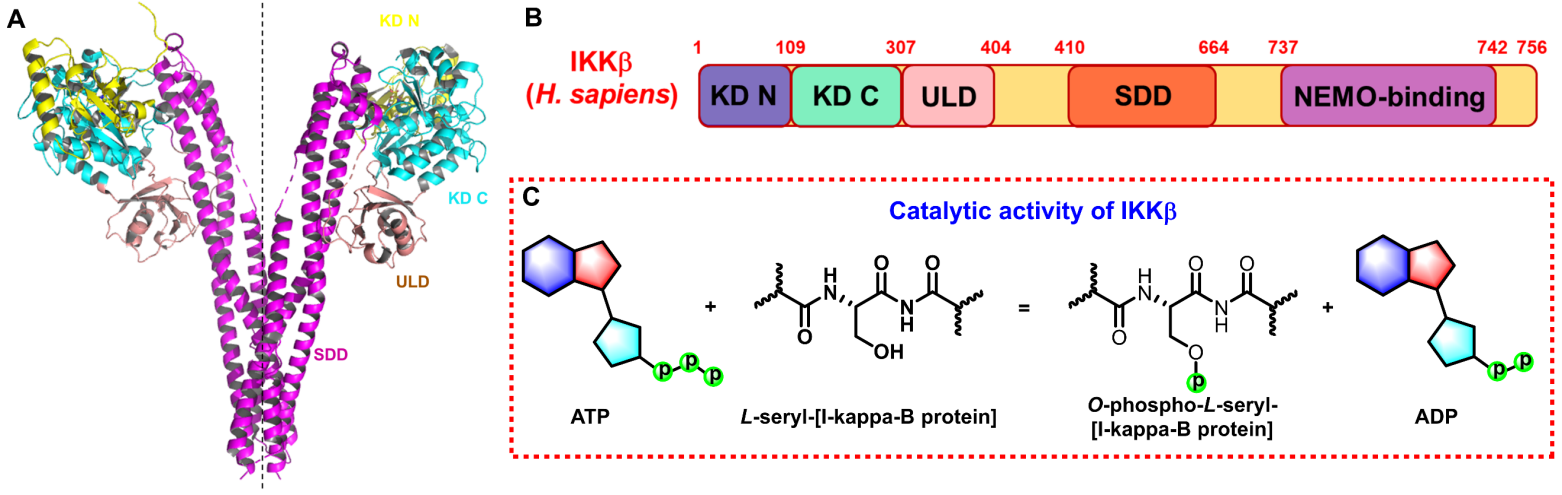
Structure and function of IKKβ. (A) 3D structure of human IKKβ dimmer (PDB: 4KIK). (B) Structural domain of human IKKβ. (C) Catalytic function of IKKβ.

The IKK complex includes IKKα, IKKβ, and IKKγ (NEMO), of which IKKα and IKKβ are the catalytic subunits, and IKKγ is the regulatory subunit 11. IKKβ is ubiquitously expressed serine-threonine protein kinase comprising of 756 amino acids, it has 52% sequence identity and 70% homology with IKKα 12, and they share highly similar domain and tertiary structure as well 13-15. As shown in **Figure 1A** (PDB: 4KIK), IKKβ contains the N-terminal kinase domain (KD N; residues 1-109), the C-terminal kinase domain (KD C; residues 110-307), the ubiquitin-like domain (ULD; residues 308-404), the scaffold dimerization domain (SDD; residues 410-664), and the NEMO-binding domain (residues 737-742, **Figure 1B**). Compared with IKKα, IKKβ has a ULD following the KD C, which is necessary for IKKβ functional activity and is important for its substrate specificity 16. IKKβ activity requires the activation of phosphorylation of Ser177 and Ser181 in the activation loop (**Figure 1C**) 17. However, until now, the exact sequence involving IKKβ activation has not been fully determined. Recent evidence for IKK complexes has demonstrated that oligomerization-mediated trans-autophosphorylation of the IKK subunit is the primary form of IKKβ activation 18. Under the action of external stimuli, TAK1 first phosphorylates Ser177 of IKKβ, which primes subsequent IKK-catalyzed autophosphorylation of Ser181 3, 8, 9. Subsequently, Ser32 and Ser36 of IκB are phosphorylated by the activated IKK complex, resulting in its ubiquitylation by the S phase kinase-associated protein 1 (SKP1)-cullin 1-F-box protein (SCF)/beta-transducing repeat-containing protein (β-TrCP) E3 ubiquitin ligase complex and degradation by the proteasome 19-21.

Single nucleotide polymorphisms (SNPs) are the most abundant form of deoxyribonucleic acid (DNA) variation in the human genome, which influences disease-related genes and non-coding RNAs via enhancing the promoter activity and specific nuclear protein-binding affinity 22. IKBKB is the gene responsible for encoding IKKβ with 21 exons localized in the chromosomal region 8p11.21. A variety of IKKβ SNPs have been reported, such as rs2272736, rs3747811, rs5029748, rs5029748, rs11986055, rs4560769, and rs6474386, and associated with the risk of hypertension, gastric and colorectal cancers, recurrent wheezing, systemic lupus erythematosus, obesity, and myelogenous leukemia 23-27. Tessier et al. found that rs3747811 served as a high risk to have an increased waist circumference in South Asian population and increase the body mass index in Caucasian population, indicating the risk between this SNP with obesity 27. Notably, SNP rs5029748 could decrease eighty percent risk for colon cancer 28. In addition, Li's group from China analyzed the association of two SNPs for IKKβ, rs12676482 and rs2272733, with systemic lupus erythematosus, and demonstrated that there is no genetic predisposition to risk of systemic lupus erythematosus in Chinese Han population 24. Therefore, the detailed relationship of SNPs with diseases requires further studies.

## The IKKβ mechanism

**Figure 2:**
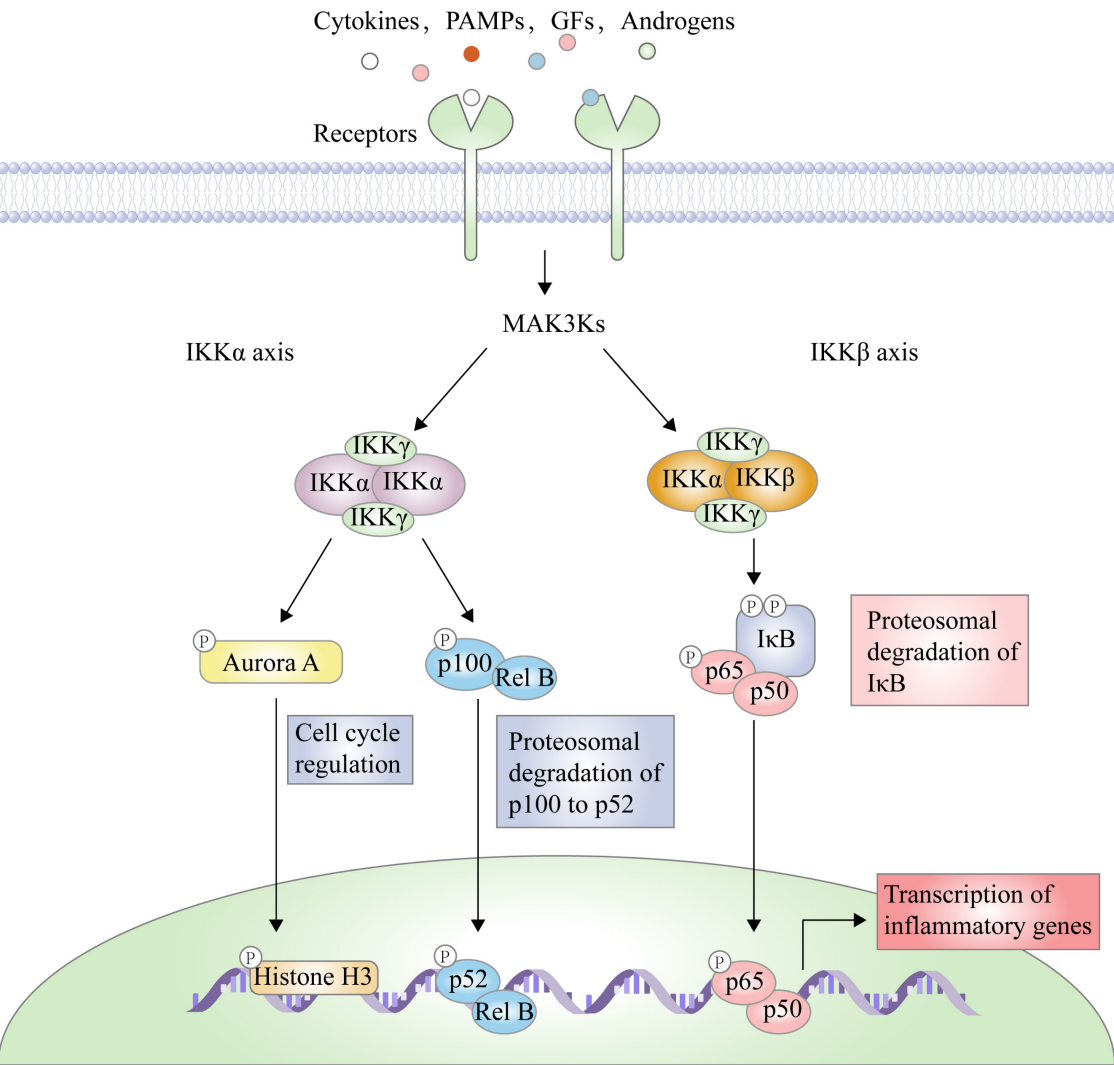
The transduction mechanism of IKKβ.

There are two principal pathways to activate NF-κB (**Figure 2**), including canonical pathway depended on IKKβ and non-canonical pathway depended on IKKα 29. IKKβ-mediated signaling pathway is activated by inflammatory cytokines or exogenous substances, such as TNF-α and LPS 30, to promote the recruitment of key enzymes, such as TAK1, MEKK1, MEKK3, NF-κB-inducing kinase (NIK), NF-κB activating kinase (NAK), and transforming growth factor kinases 31. For example, under the IL-1 stimulation, the protein domain of TNF receptor-associated factor 6 (TRAF6) finger catalyzes the polyubiquitination of Lys63-linked NEMO, and mediates the binding of TAK1 and TAB2/3 through the interaction between ubiquitin chains, specifically activating IKKβ through the phosphorylation of Ser177 and Ser181 32. Subsequently, Ser32 and Ser36 of IκB are phosphorylated by the activated IKK complex, resulting in its ubiquitylation by the SCF/β-TrCP E3 ubiquitin ligase complex and degradation by the proteasome 19-21, 33. Because IκB normally binds to NF-κB, the latter's nuclear localization sequence (NLS) is masked, resulting in its main location in the cytoplasm, while removal of IκB exposes the masked site to induce the nuclear translocation of NF-κB, making it bind to the promoter regions of its downstream genes 34, such as cyclooxygenase-2 (COX-2), IL-6, and inducible nitric oxide synthase (iNOS). Furthermore, activated IKKβ mediates the Ser536 phosphorylation of NF-κB p65 to initiate reverse transcription, resulting in increased transcriptional activation 35, 36. In the non-canonical pathway, IKKα activates NF-κB as well. Although IKKα and IKKβ have contain similar structural domains and possesses 70% homology, the kinase activity of IKKβ towards IκB is 20-50-fold higher than that of IKKα 37, therefore, IKKβ have a higher status in activation of NF-κB 38.

IKKβ-mediated NF-κB pathway is a key signal transduction pathway involved in inflammatory response, angiogenesis, invasiveness, metastasis, and immune escape 39-46. After this signaling pathway is activated by inflammatory factors, it promotes the expression of inflammatory factors at the transcriptional level as well, thus forming a loop and producing an amplification effect to aggravate inflammation response 47-51. In the case of the colitis-associated cancer (CAC) mouse model, IKKβ genetic deletion in intestinal epithelial cells greatly reduces the incidence of tumors through the increase of apoptosis 52, while its KO in the macrophage significantly reduces tumor size by suppressing the expression of pro-inflammatory factors 53, 54. Therefore, IKKβ has be considered a potential target to develop drugs in the treatment of inflammation and caners.

## Biological potentials for IKKβ inhibition

IKKβ serves as a role in biological functions, such as immunity and inflammation, because of IKKβ responsible for NF-κB pathway. Accumulating evidence has demonstrated that caners, CNS, and metabolic diseases, including pancreatic cancer, Parkinson's disease (PD), diabetes, non-alcoholic fatty liver disease (NAFLD), are associated with IKKβ 55-57. In addition, chemical inhibition of IKKβ or its genetic KO has beneficial effects to block NF-κB mediated above-mentioned diseases 3, 8, 9.

### Cancers

Cancer is a terrible disease that has existed for more than 200 million years and first appeared in humans more than one million years ago. Cancer, different from infectious, parasitic, and other diseases caused by many environmental factors, is mainly caused by abnormal changes of its own normal cells under the long-term action of internal and external factors 58, 59. IKKβ is overexpressed in tumors, including pancreatic, breast, ovarian, lung, myeloma, rectal, and leukemia cancers, and IKKβ-mediated NF-κB pathway is a key signal transduction pathway involved in the occurrence and development of tumors 39. The phosphorylation of IKKβ at Ser177 and Ser181 promotes the IκBα phosphorylation to trigger the ubiquitination and degradation of the latter to worsen the NF-κB cascade 3, 8, 9. Accumulating evidence demonstrates that IKKβ genetic KO and its chemical inhibition both block the NF-κB transduction to alleviate the tumor growth, such as pancreatic, colorectal, lung, and myeloma cancer cells 60-62. Therefore, IKKβ plays a critical role in the treatment of cancers *via* blocking the NF-κB cascade.

Pancreatic cancer, first described in 1761, belongs to the member of malignant tumors with a high mortality 55. Although enhancing survival rates of other cancers, that of pancreatic cancer still remains invariability since 1960s. Because of its insidious onset, the early detection is difficult, and symptoms generally appear in the middle and late stages, such as abdominal pain and jaundice, therefore, most treatment regimens are ineffective 55. The present series of studies provide evidence that IKKβ is overexpressed in pancreatic cancer cells, such as Panc-1, MIA-PaCa-2, and AsPc-1 63, 64, revealing its role in pancreatic cancer. Wu and co-workers treated pancreatic cancer cells AsPc-1 with apigenin and found its potential in the inhibition of the growth of pancreatic cancer cells 63. Subsequent experiments demonstrated that apigenin inhibited the IKKβ activity to promote the cleaved caspase 3 expression, allowing the apoptosis of pancreatic cancer cells 63. Similarly, Tong et al. demonstrated that an IKKβ inhibitor emodin suppressed the growth of gemcitabine resistant pancreatic cancer cell through apoptosis 64. Inhibition of IKKβ by a quinoxaline urea inhibitor, synthesized by Radhakrishnan et al. in 2021, also displayed the antiproliferative effect against pancreatic cancer cells T3M4 and MiaPaCa2 65.

**Figure 3:**
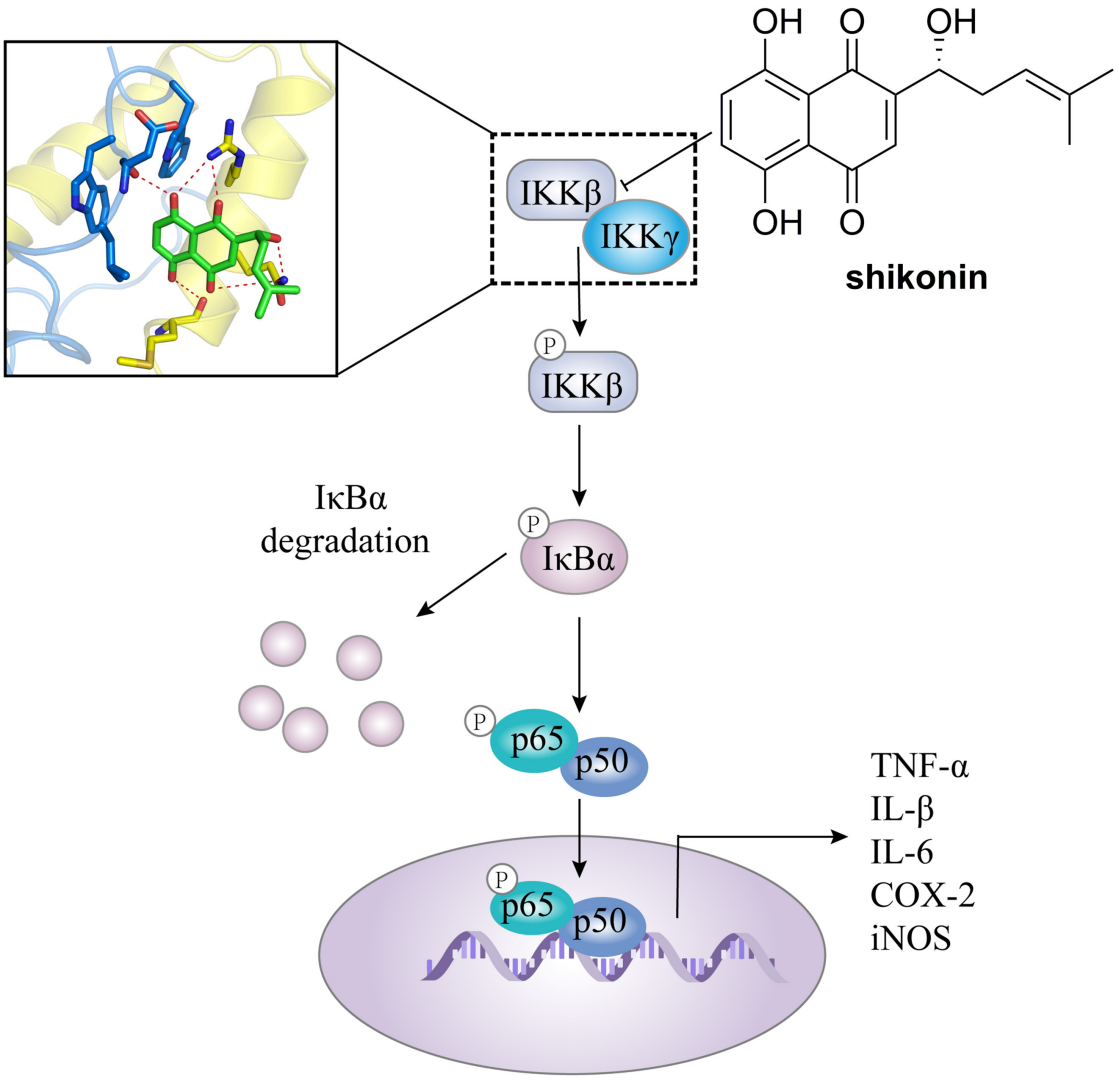
The mechanism of action of shikonin through suppressing the binding of IKKβ with IKKγ in against colorectal cancer.

In addition, IKKβ inhibitors, e.g. abietic acid, alantolactone (**16**), MLN120B, and shikonin, have been used in the treatment of other cancers as well 60, 66, 67. For colorectal cancer, Yu et al. found the antitumor effect of shikonin against colorectal cancer cells. Shikonin effectively blocked the IKKγ/IKKβ complex formation *in vitro* and *in vivo* via preventing the IKKγ-binding domain (NBD) of IKKβ from entering the hydrophobic pocket of IKKγ (**Figure 3**), resulting the inhibition of proliferation, migration, and invasiveness 60.

### CNS diseases

CNS diseases are one of the major problems threatening human health, and out of balance of the redox system and intense neuroinflammation are the major causes of CNS diseases 68. In the CNS immune system, activation of microglial cells results in the neuroinflammation responsible for the elimination of pathogens, toxic components, and dead cells. Activated IKKβ promotes the NF-κB cascade to magnify the activization of microglial cells, allowing the release of pro-inflammatory factors and chemokines to worsen CNS diseases, therefore, suppressing the IKKβ activity is considered the strategy for the treatment of this type disease.

PD is a neurodegenerative disease characterized by the loss of dopamine neurons in substantia nigra, which often occurs in the elderly 69. The current drugs for treating this disease can only control its further deterioration but accompanied by other adverse reactions. Recently, it has been reported that IKKβ-mediated NF-κB pathway also plays a key role in regulating neuroinflammation for PD. In LPS- or 1-methyl-4-phenyl-1,2,3,6-tetrahydropyridine (MPTP)-mediated PD animals, the IKKβ activity is increased in substantia nigra because of the overexpression of its phosphorylation 70-72, which significantly promoted LPS-induced neurotoxicity 71, such as dopaminergic and tyrosine hydroxylase positive cell death, while its genetic abolishment or inhibition of IKKβ attenuated the neuroinflammation 70, 73-76. For example, Zhang et al. has reported that administration of IKKβ inhibitors BAY-65-1942 inhibited the microglial activation, attenuated the loss of dopamine neurons, and enhanced levels of neurotransmitters 71, and the similar result was observed after administration of IKKβ inhibitors, such as BAY-65-1942, metformin, and ginsenoside Rb1, and peptide 70, 74, 75. Accordingly, IKKβ functions as a therapeutic target for PD.

Alzheimer's disease (AD) is a neurodegenerative disease represented by impaired learning, memory, and cognitive abilities 77. In AD, the extracellular amyloid beta (Aβ) deposition leads to the activation of microglia to trigger the neuroinflammation that causes the neuronal death and cognitive deficits 78. Accumulating clinical evidences demonstrate that 20% of AD patients are associated with severe neuroinflammation 77, 79, meanwhile regulation of neuroinflammation is closely related to the immune and nervous system serves as a therapeutic strategy for AD 78. A large number of data reasoned the activation of IKKβ/NF-κB by stimulators (e.g. TNF-α and IL-6) in neurons, microglia, and astrocytes to trigger the malignant cascade of AD 78, 80, 81. Meanwhile, increasing of preclinical studies focused on IKKβ demonstrated the relationship between IKKβ and AD 82. A study by Liu et al. suggested that the IKKβ deficiency in myeloid cells attenuated AD-related symptoms and pathology, such as cognitive deficits, inflammation, and Aβ load, by enhancing microglial and macrophage recruitment towards Aβ deposits and internalization 83. Karpagam et al. has reported that celastrol (**26**), a natural product from *Tripterygium wilfordii*, inhibited the IKKβ activity to attenuate the Aβ cytotoxicity, triggering the neuroprotective effect in AD 56. In addition, its analogue depended on the inhibition of IKKβ not only displayed therapeutic effects in AD, but also in metabolic diseases, amyotrophic lateral sclerosis, PD, and Huntington's disease 84-86.

Spinal cord injury (SCI) is a permanent motor and sensory deficits characterized by the invasive degeneration of the spinal cord tissue caused by the mechanical trauma to the neurons and surrounding vasculature 87. Recently, Lee and co-workers revealed the role of IKKβ in neuroinflammation, one of the mechanisms of secondary SCI 88, 89. This study found that IKKβ KO reduced the infiltration of neutrophils and macrophages and the pro-inflammatory factor expression through suppressing the myeloid cell activation, allowing the attenuation of neuronal loss and behavioral deficits in SCI 89. Consistently, emerging evidence has indicated that targeting IKKβ with drugs or microRNAs alleviates the course of SCI 90-94. For example, Li et al. demonstrated that tamoxifen administration inhibited the infiltration of inflammatory cells and neuron apoptosis via regulating the IKKβ pathway 94. In addition, inhibition of IKKβ alleviated IL-1β-mediated NF-κB activation to trigger the blocking of DNA binding, resulting in the remission of tactile and cold allodynia 95.

### Metabolic diseases

Metabolic diseases are becoming increasingly common in modern society, such as diabetes, NAFLD, and non-alcoholic steatohepatitis (NASH) due to people's lifestyles and irregular circadian rhythms 96. Obesity is a low-grade sustained inflammatory disease, affecting about 30% of population all over the world 97, 98. Furthermore, obesity has become an inducement to cause other diseases, and increases the risk of diabetes, NAFLD, and NASH under the stimulation of the chronic inflammation as well 98, 99. Recently, increasing studies have demonstrated the effect of IKKβ in the obesity-mediated inflammation 100. Douglass et al. found that special IKKβ genetic deletion in the astrocyte attenuated phenotypes of obesity, such as weight gain, glucose intolerance, and insulin resistance, in obesity mice 100. Similarly, IKKβ inhibitor IMD-0354 not only promoted the adiponectin secretion *in vitro*, but also attenuated insulin resistance and enhanced plasma levels of adiponectin in the high-fat diet (HFD)-induced animal model 101. In addition, SA18 and SA32, other IKKβ inhibitors, displayed similar effects in the obesity 102.

As a major public health issue, diabetes belongs to one of the family metabolic diseases characterized by hyperglycemia caused by insulin resistance 103. A previous study by Salem et al. has reported that the specific IKKβ activation in β cells could cause to immune-mediated diabetes 104. Furthermore, a great body of evidences have demonstrated that IKKβ genetic KO or its inhibitors displayed the therapeutic effect on diabetes 105-107. For example, the IKKβ overexpression could suppress the insulin pathway, and IKKβ genetic deletion alleviated the course of insulin resistance in the obese animal model, such as HFD-induced and *Lep^ob/ob^* obese mice 108. Collectively, inhibition of IKKβ by salicylate or celastrol (**26**) decreased insulin resistance and lipid abnormalities and increased the adiponectin level to attenuate the adiposity 108, 109.

Besides, emerging evidence has indicated the overexpression of IKKβ in liver diseases, including NAFLD, NASH, and liver fibrosis 110. NAFLD is a series of liver diseases to cause hepatic steatosis and death of liver cells, eventually resulting in cirrhosis and liver failure 57. Over past few decades, the prevalence of NAFLD had increased year by year, and the prevalence of obesity and diabetes had increased as well. Because NAFLD was related to obesity and diabetes, it had attracted widespread attention 111-113. Although the treatment of NAFLD had made great progress during the past decades, the mechanism of its development is still unclear 57, 114. So far, two hypotheses on the mechanism of NAFLD are approved by academic community. One is the imbalance of fatty acid metabolism, which leads to the accumulation of triglycerides in the liver and steatosis; other is oxidative or metabolic stress and cytokine unbalance 114. Dou et al. found that the IKKβ S-glutathionylation by glutathione disulfide amplified TNF-α mediated hepatotoxicity, revealing that IKKβ *S*-glutathionylation was the potential mechanism of NAFLD 57.

### Others

Chemical inhibition of IKKβ is to the benefit of many other diseases, such as osteolysis, arthritis, premature birth, radiation injury, and allergy 59, 115-117. McIntyre and co-workers investigated the protective effect of BMS-345541, an IKKβ inhibitor, and found that it could suppress the IL-1β level to attenuate inflammation and joint destruction, contributing to alleviate collagen-induced arthritis in the mouse model 117. The similar result was observed in the rheumatoid arthritis through inhibiting the IKKβ activity by 4-[6-(cyclobutylamino)imidazo[1,2-*b*]pyridazin-3-yl]-2-fluoro-*N*-([(2*S*,4*R*)-4-fluoropyrrolidin-2-yl])methylbenzamide 118. Furthermore, the selective IKKβ inhibitor SC-514 inhibited the IκBα degradation to inactivate the NF-κB signal, further triggering to impair RANKL (receptor activator of nuclear factor-κB ligand)-induced osteoclastogenesis 119, which was further supported by Thummuri's study showing that inhibition of IKKβ by abietic acid attenuated RANKL-induced osteoporosis 120. The abovementioned findings all suggested that that targeting IKKβ was a potential treatment for osteoclast-related disorders. In addition, some IKKβ inhibitors, including tetrandrine, IMD-0560, and IMD-0354, were applied for the treatment of preterm delivery, radiation damage, and hyperalgesia 59, 115, 116. So far, many IKKβ inhibitors displayed significant therapeutic effects in pre-clinical animal experiments, and some inhibitors, such as MLN-120B, IMD-2560, and SAR-113945, have finished the phase I or II clinical trial for the safety, tolerability, and pharmacokinetics. However, IKKβ deficient mice die on 14^th^ day of gestation because of massive apoptosis of hepatocytes, therefore, these clinical trials have to be terminated 38, 121. Therefore, discovery of new IKKβ inhibitors has sharply declined in the recent decade 121.

## Targeting IKKβ with pharmacological small-molecule compounds

IKKβ potentials have been paid more and more attentions in the prevention and treatment of various diseases as the discovery of the first IKKβ inhibitor SPC839 (**57**) in 2001, therefore, its development has become a hot topic. To date, IKKβ inhibitors have been found from various pathways, including natural products and chemical synthesis, herein, advances of IKKβ inhibitors are summarized.

### Natural IKKβ inhibitors

#### Sesquiterpenoids

**Figure 4:**
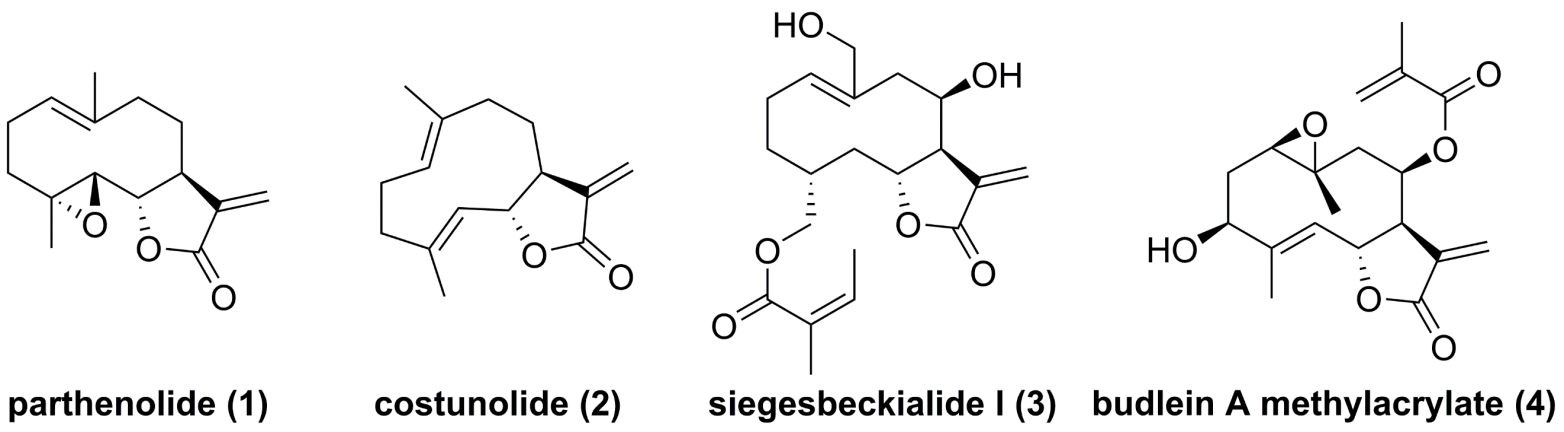
Chemical structure of parthenolide (**1**), costunolide (**2**), siegesbeckialide I (**3**), and budlein A methylacrylate (**4**).

**Figure 5:**
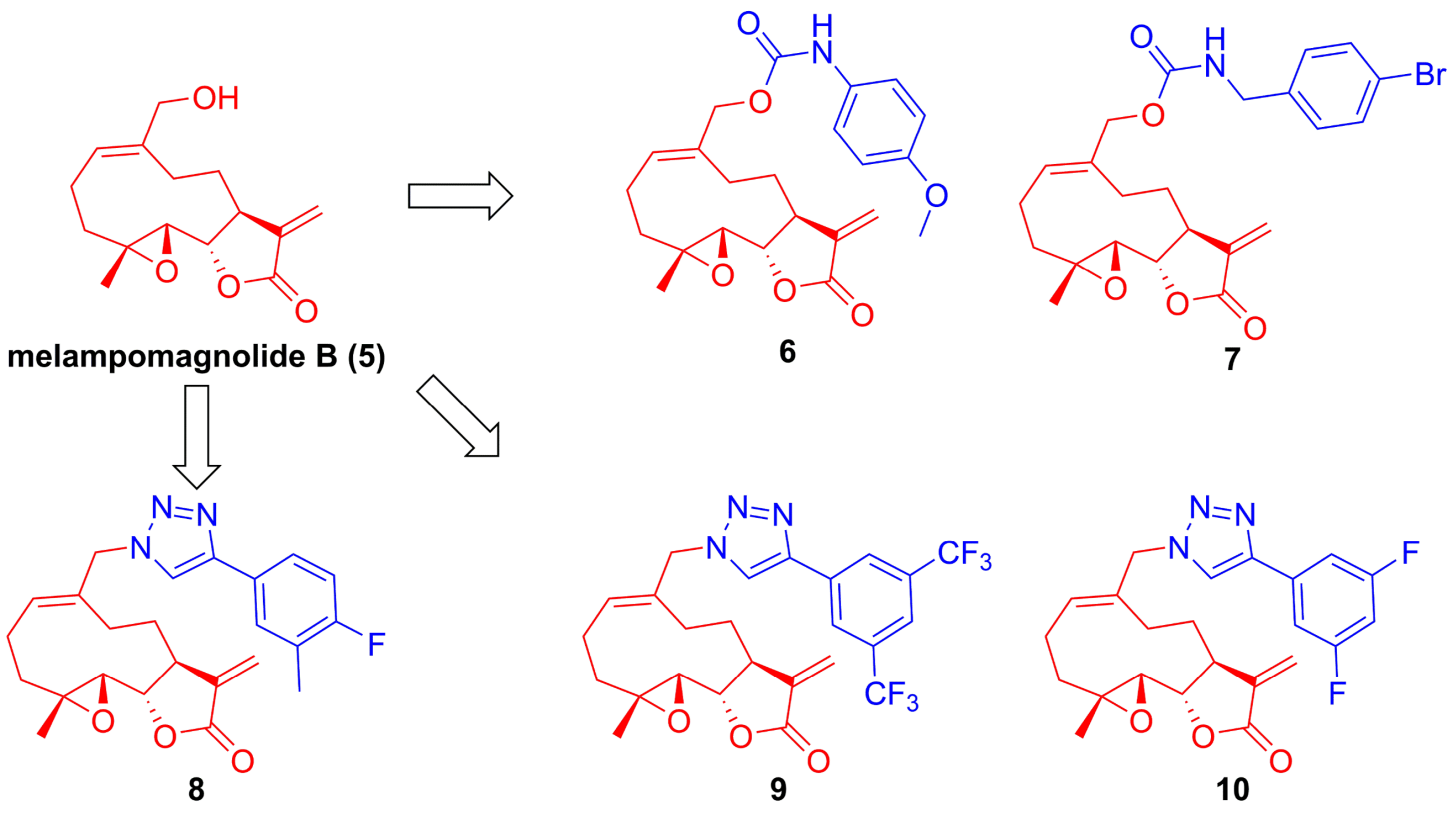
Structural modification of melampomagnolide B (**5**)

Parthenolide (**1**, **Figure 4**), naturally occurred in *Tanacetum parthenium*, belongs to the family of 5,10-*seco*-eudesmane type sesquiterpenoids, and its inhibitory effect towards IKKβ was first reported by Benjamin in 2001 122, 123. Researchers from China also found that a naturally occurring parthenolide analogue, costunolide (**2**), could covalently bind to Cys179 of IKKβ, and inhibit its activity, triggering its protective effect toward atherosclerosis in HFD-fed *ApoE*^-/-^ mice 124. *Sigesbeckia glabrescens* is a species of the genus *Sigesbeckia* first recorded in Xinxiu Herba written by Su Jing (Tang Dynasty) 125. Based on its chemical constituents, Gao and colleagues obtained twenty-four sesquiterpenoids, including ten new compounds. They found that siegesbeckialide I (**3**, **Figure 4**) significantly displayed an inhibitory effect against IKKβ via covalently binding to amino acid residue Cys46 in the KD N region of IKKβ with the olefinic bond of an *α*,*β*-unsaturated lactone 125. Budlein A methylacrylate (**4**), a natural sesquiterpenoid isolated from the genus *Helianthus*, displayed an anti-triple-negative breast cancer effect 126. Based on the result of pull down depended on its probe biotinylated **4** (Bio-**4**, **Figure 4**), Wang et al. found that **4** could bind to IKKβ through the covalently binding of Cys179 in IKKβ 126. Subsequently, Crooks's group demonstrated the potential of a parthenolide analogue, melampomagnolide B (**5**, **Figure 5**), towards IKKβ, and optimized its structural to afford a library of its analogues, such as **6-10** 127-129. Because of their binding to the IKKβ ULD, **6** and **7** displayed significantly inhibitory effects 127.

**Figure 6:**
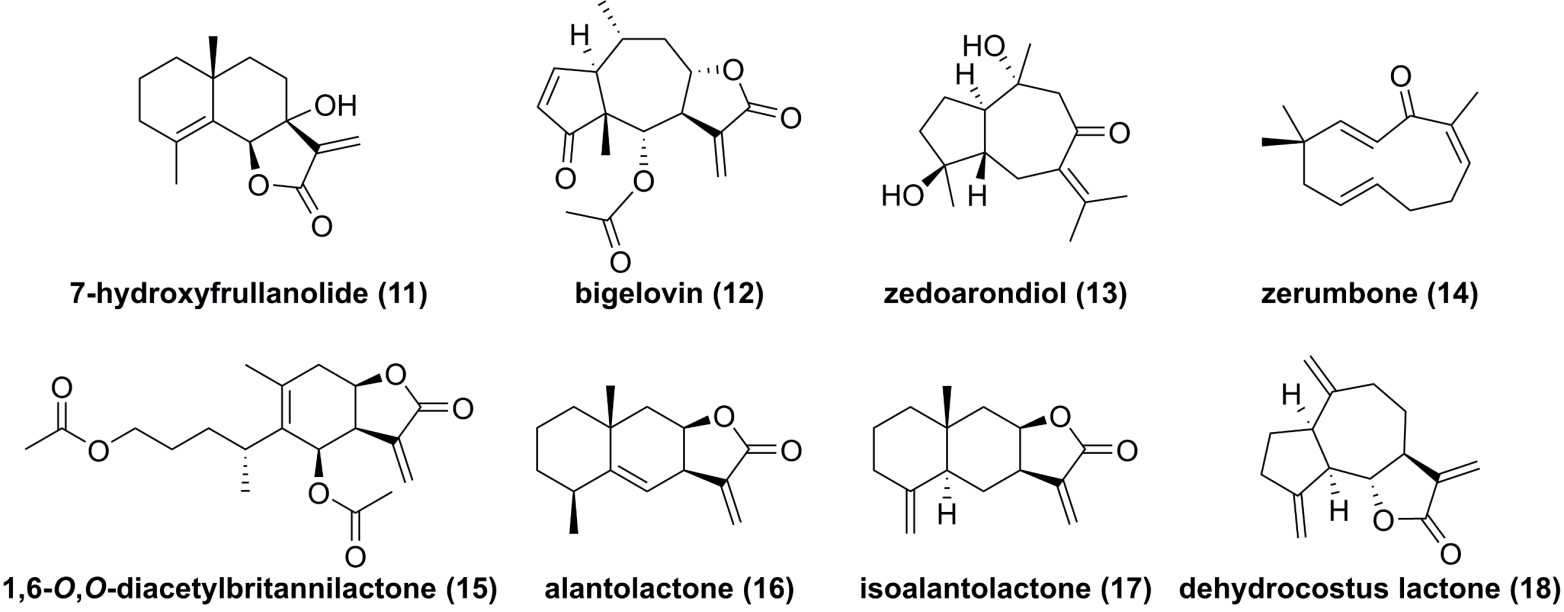
Chemical structure of sequiterpnoids **11-18**.

7-Hydroxyfrullanolide (**11**, **Figure 6**), isolated from *Sphaeranthus indicus*, is a sesquiterpene lactone with a 6/6/5 skeleton and possesses various bioactive activities 130. Fonseca et al. found that 7-hydroxyfrullanolide (**11**) could suppress LPS-mediated NF-κB activation 130. In-depth investigation on its mechanism revealed its anti-inflammatory effect depended on IKKβ, which was supported by the IKKβ kinase assay 130. Feng and co-workers demonstrated that bigelovin (**12**), a pseudoguaiane-type sesquiterpenoid from the genus *Inula*, could induce the IKKβ degradation to suppress the NF-κB pathway, thus causing apoptosis of colon cancer cell 131. Recent studies reported potentials of zedoarondiol (**13**), zerumbone (**14**), and 1,6-*O*,*O*-diacetylbritannilactone (**15**) against IKKβ as well 132-134. In addition, our group also investigated chemical constituents of *Inula helenium* known as “Tu mu xiang” in Chinese, and found that alantolactone (**16**), isoalantolacton (**17**), and dehydrocostus lactone (**18**) exerted antitumor activities against glioblastoma through targeting IKKβ 66, 135, 136.

**Figure 7:**
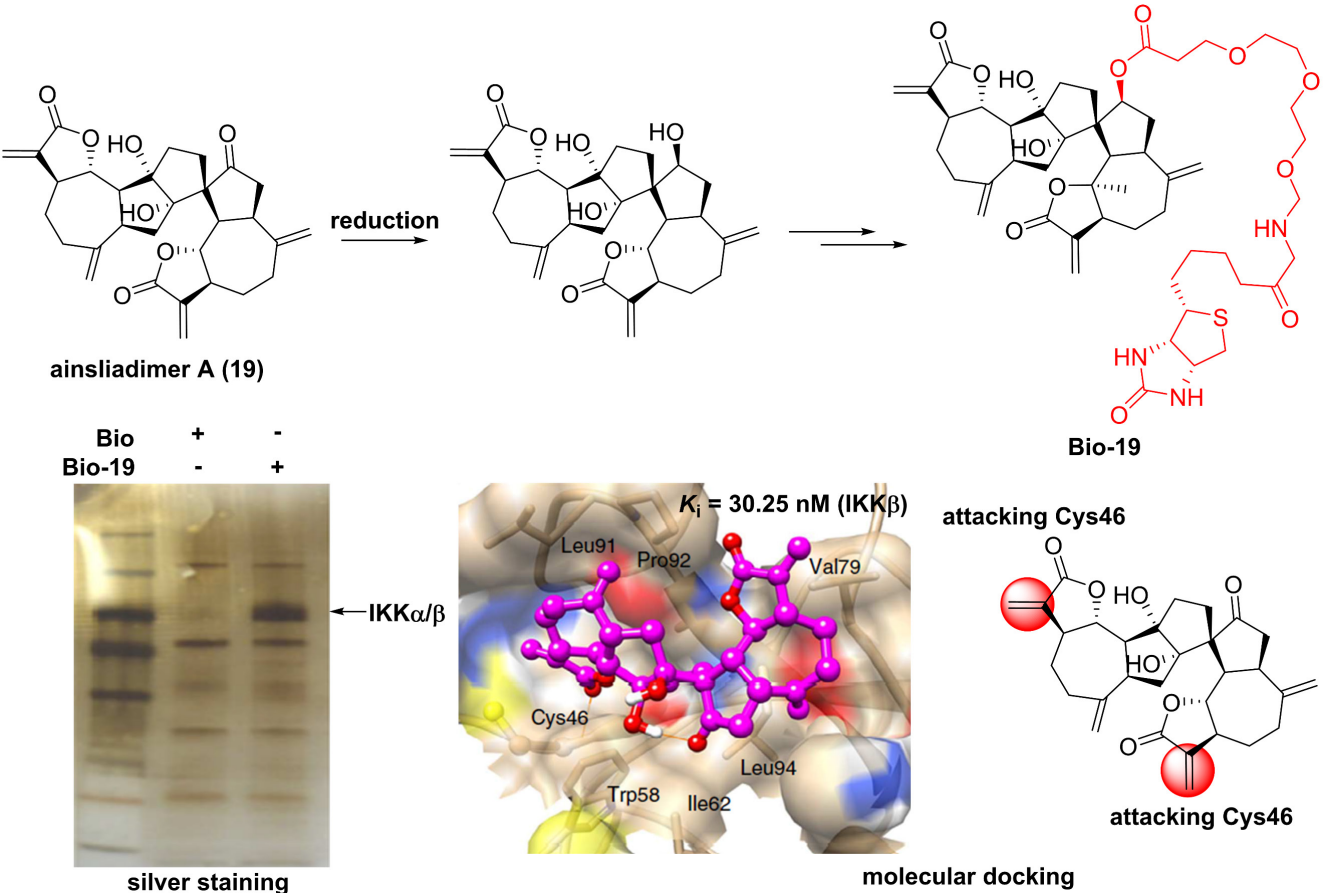
Chemical structure of ainsliadimer A (**19**) and the discovery of its covalently binding target.

For the sesquiterpenoid dimer, Lei's group found that ainsliadimer A (**19**, **Figure 7**), isolated from *Ainsliaea macrocephala*, displayed an anti-inflammatory potential in Raw264.7 cells after exposure to LPS 137. In order to reveal its direct cellular target, Lei and co-workers reduced the ketone carbonyl moiety of ainsliadimer A (**19**) and linked a biotin to afford a probe Bio-**19**. Using this probe to fish the binding proteins, Lei et al. found that ainsliadimer A (**19**) could selectively suppress IKKα/β via covalently binding to Cys46 in the activation loop of IKKα/β, meanwhile ainsliadimer A (**19**) had a good affinity (*K*_i_ = 30.25 nM) and specific reactivity (*K*_inact_ = 7.74 min^-1^) with IKKβ 137.

#### Diterpenoids

**Figure 8:**
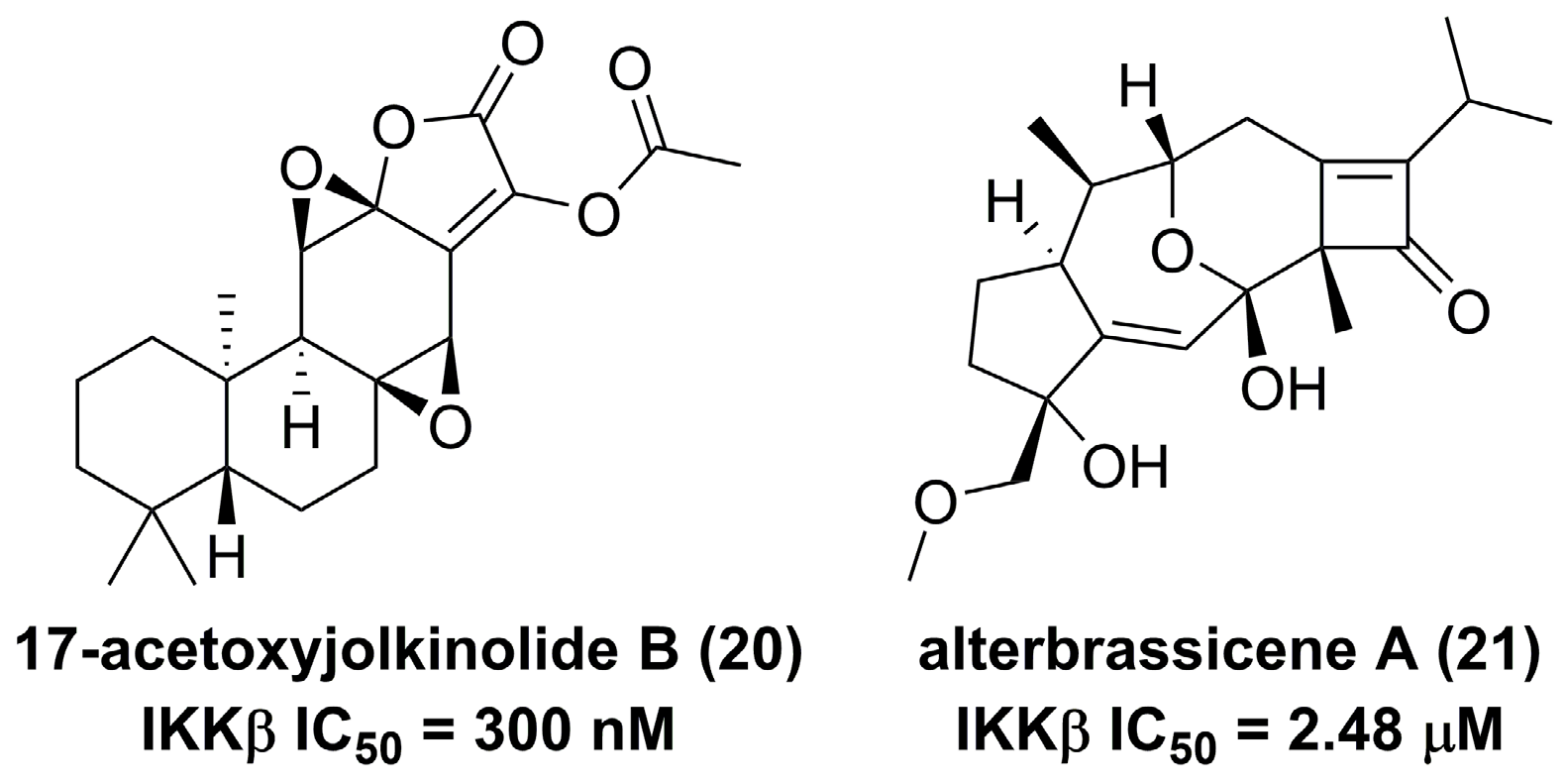
Chemical structures of diterpenoids **20** and **21**.

**Figure 9:**
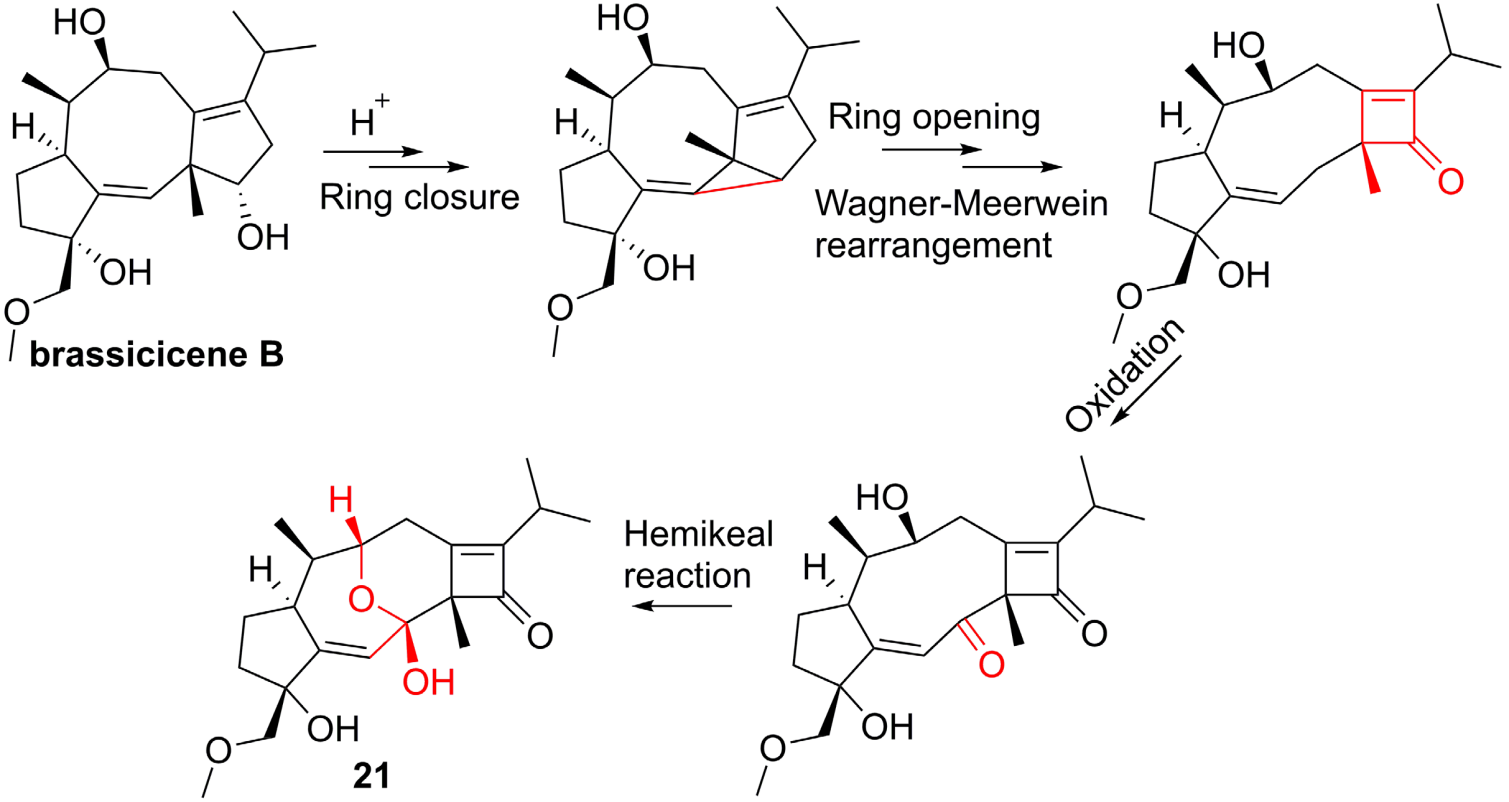
Plausible Biosynthetic Pathway for **21**.

**Figure 10:**
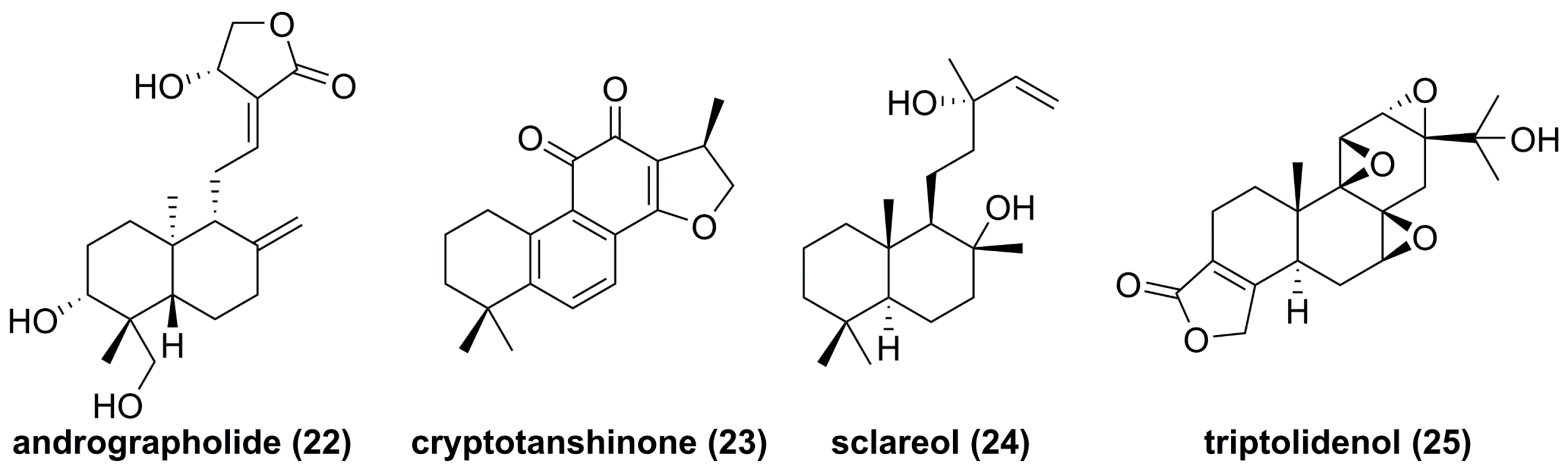
Chemical structures of diterpenoids **22-25**.

The root of dried *Euphorbia fischeriana* Steud is a traditional Chinese medicine first recorded 2,000 years ago, known as Langdu in Chinese, to treat cancer, edema, and ascites 138. Yan and co-workers afforded seven diterpenoids from *E. fischeriana,* and found that 17-acetoxyjolkinolide B (**20**, **Figure 8**) displayed a remarkable inhibitory activity against TNF-α-medicated NF-κB activation. The investigation on its action mechanism demonstrated that 17-acetoxyjolkinolide B (**20**) could suppress the phosphorylation of TNF-α-medicated IκB and the nuclear translocation of p65 via inhibiting IKKβ (IC_50_ = 300 nM) 139. In addition, a study by Hu et al. focused on fusicoccanes from *Alternaria brassicicola* found alterbrassicene A (**21**) sharing a 5/9/4-fused carbocyclic skeleton with a rare fused 2-cyclobuten-1-one motif 140, and its biosynthetic pathway was proposed as shown in **Figure 9**. It is worth noting that alterbrassicene A (**21**) displayed an inhibitory effect against IKKβ (IC_50_ = 2.48 μM), and it could bind to KD N and C lobes of IKKβ through hydrogen bond interactions with Leu21 and Arg31 140. Andrographolide (**22**, **Figure 10**) is a characteristic *ent*-labdane diterpenoid of *Andrographis paniculata* (Burm. f) Nees, a traditional medicinal plant in China, and possesses multiple bioactivities, such as antitumor and anti-inflammatory effects 141. Chao and co-workers demonstrated that andrographolide (**22**) inhibited the IKKα/β activity to reduce the increase of TNF-α mediated ICAM-1 expression and the activation of TNF-α mediated NF-κB 141. Cryptotanshinone (**23**), a diterpene quinone from traditional Chinese herb *Salvia miltiorrhiza*, has multiple potentials on various diseases 142. In 2016, Wu et al. reported its anticancer activity towards acute lymphoblastic leukemia, and revealed its mechanism involved in the binding to the ATP binding region of IKKβ as same as MG132 (an IKKβ inhibitor) through interactions of Val29, Glu97, Tyr98, Cys99, Glu100, and Gly102 142. Phytochemical investigation focused on *Sagittaria trifolia* resulted in 11 diterpenoids, and subsequent study on anti-proliferative effects demonstrated the inhibitory potential of sclareol (**24**) toward IKKβ 143. In addition, triptolidenol (**25**) was reported as well, and the result of molecular docking demonstrated its mechanism of action towards IKKβ 144.

#### Triterpenoids

**Figure 11:**
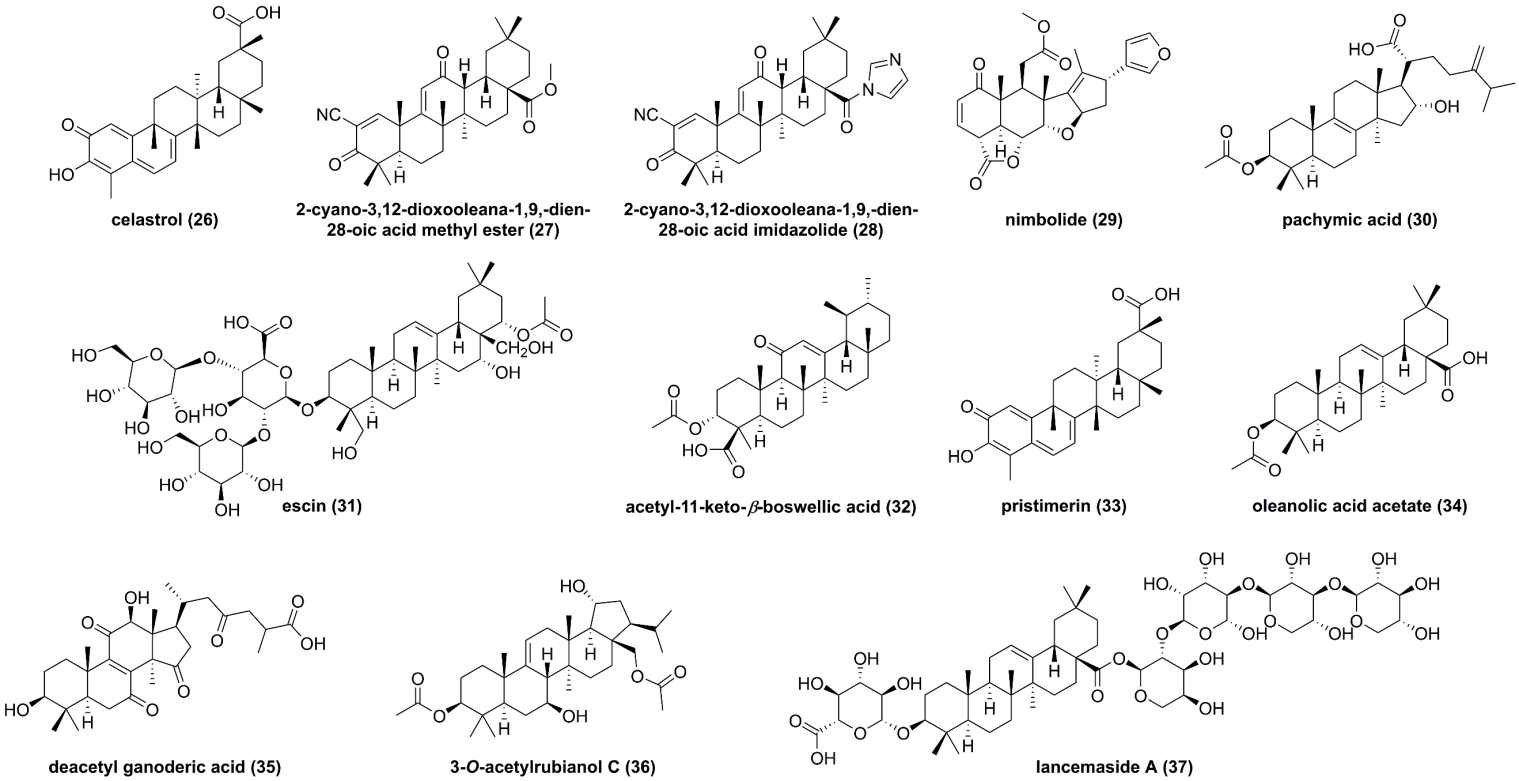
Chemical structures of diterpenoids **26-37**.

Triterpenoids are common type compounds in natural products, so far, some of triterpenoids have been reported from *Celastrus orbiculatus*,* Azadirachta indica, and Codonopsis lanceolata.* Celastrol (**26**, **Figure 11**) is a quinone methide triterpenoid isolated from *Celastrus orbiculatus*, and showed inhibitory effects against TNF-α-, IKKβ-, or MEKK1-mediated NF-κB activation on the basis of NF-κB luciferase activity 145. Subsequently, Lee and colleagues found that celastrol (**26**) could dose-dependently suppress the IKKβ activity, and the mutation of Cys179 in the activation loop of IKKβ abolished its effect, indicating that it targeted amino acid residue Cys179 of IKKβ to inhibit the activation of NF-κB 145. In 2006, Ahmad et al. also reported that 2-cyano-3,12-dioxooleana-1,9,-dien-28-oic acid methyl ester (**27**), a pentacyclic triterpene, interacted with Cys179 to inhibit the IKKβ activity 146. Subsequent studies focused on the structural modification of **27** afforded a new synthetic triterpenoid inhibitor 2-cyano-3,12-dioxooleana-1,9,-dien-28-oic acid imidazolide (**28**) 147. In 2010, Gupta et al. found that the characteristic constituent of *Azadirachta indica*, nimbolide (**29**), promoted the apoptosis induced by chemotherapeutic agents in tumor cells 148.

The study on the mechanism of action of **29** demonstrated that it could modify Cys179 of IKKβ to suppress the IKKβ activity, which was supported by amino acid mutation Cys179Ala 148. In the past decade, pachymic acid (**30**) from *Poria cocos*, escin (**31**), acetyl-11-keto-*β*-boswellic acid (**32**) from the genus *Boswellia*, pristimerin (**33**) from *Menispermum dauricum*, oleanolic acid acetate (**34**) from *Vigna angularis*, deacetyl ganoderic acid (**35**) from *Ganoderma lucidum*, 3-*O*-acetylrubianol C (**36**) from *Rubia philippinesis*, and lancemaside (**37**) from *Codonopsis lanceolata* inhibited the IKKβ activity or regulated the phosphorylation of IKKβ 149-155. For example, oleanolic acid acetate (**34**) exerted an anti-inflammatory effect via inhibiting IKKα/β to reduce the production of embryonic alkaline phosphatase and pro-inflammatory cytokines, including MCP-1, IL-1, IL-8, VCAM-1, and ICAM-1 152. In addition, lancemaside A (**37**), a pentacyclic triterpenoid glucose isolated from *Codonopsis lanceolata*, inhibited the LPS-mediated inflammatory response in Raw264.7 cells through blocking the IKKβ activation 156.

#### Flavonoids

**Figure 12:**
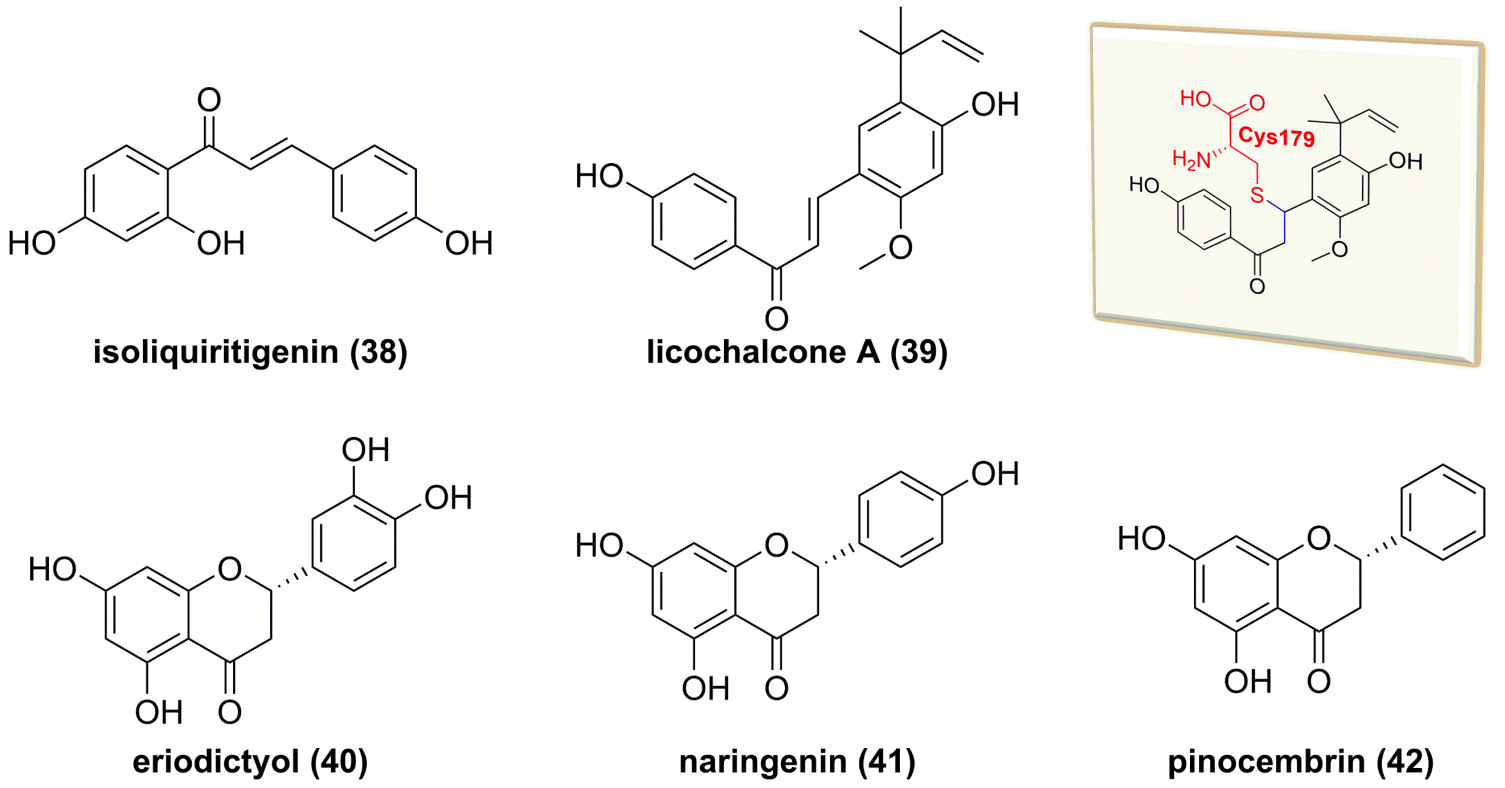
Chemical structures of diterpenoids **38-42** and interaction of **39** with IKKβ.

*Glycyrrhiza inflata* is a traditional Chinese medicine to treat inflammatory related diseases, and its major chemical constituents are flavonoids and triterpenoids 157. Isoliquiritigenin (**38**, **Figure 12**) and licochalcone A (**39**), isolated from the genus *Glycyrrhiza*, belong to the chalcone family and possess multiple bioactive activities, such as anti-inflammatory and anti-tumor effects 158, 159. A study by Yan and co-workers reported the inhibitory potential of isoliquiritigenin (**38**) against human T lymphocyte activation 158. Its mechanism involved in the covalently binding Cys46 of IKKβ via Michael addition because of the presence of an *α*,*β*-unsaturated ketone moiety in isoliquiritigenin (**38**) 158. Similarly, Megumi et al. found that licochalcone A (**39**) inhibited IKK complex activation and IκB degradation via the covalently binding to amino acid residue Cys179 of IKKβ 159. Additionally, some of dihyflavones, such as erioddictyol (**40**), naringenin (**41**), and pinocembrin (**42**), could interact with amino acid residues Thr23, Glu97, Cys99, and Asp166 in the ATP binding domain through hydrogen bonds to suppress the IKKβ activity 160.

#### Alkaloids

**Figure 13:**
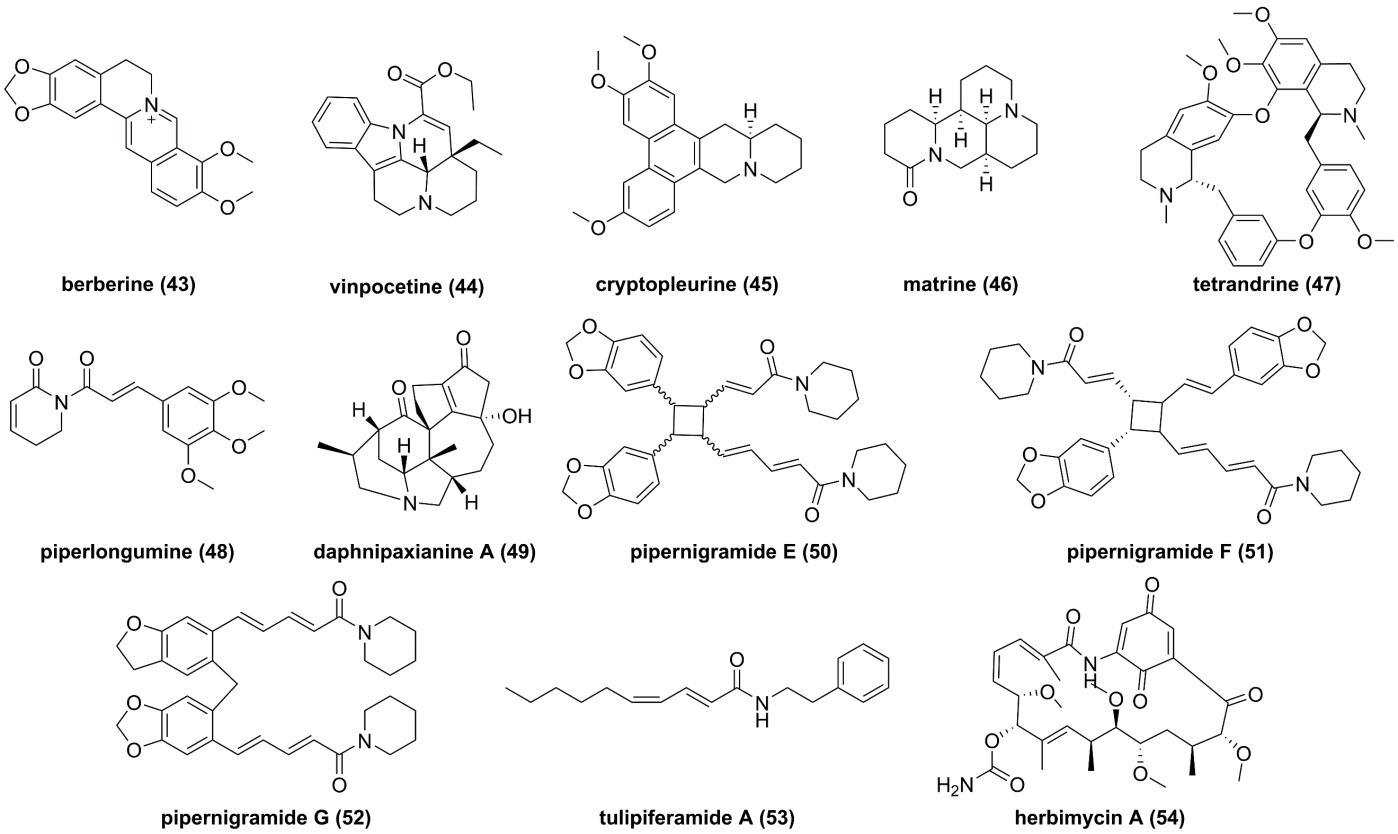
Chemical structures of diterpenoids **43-54**.

**Figure 14:**
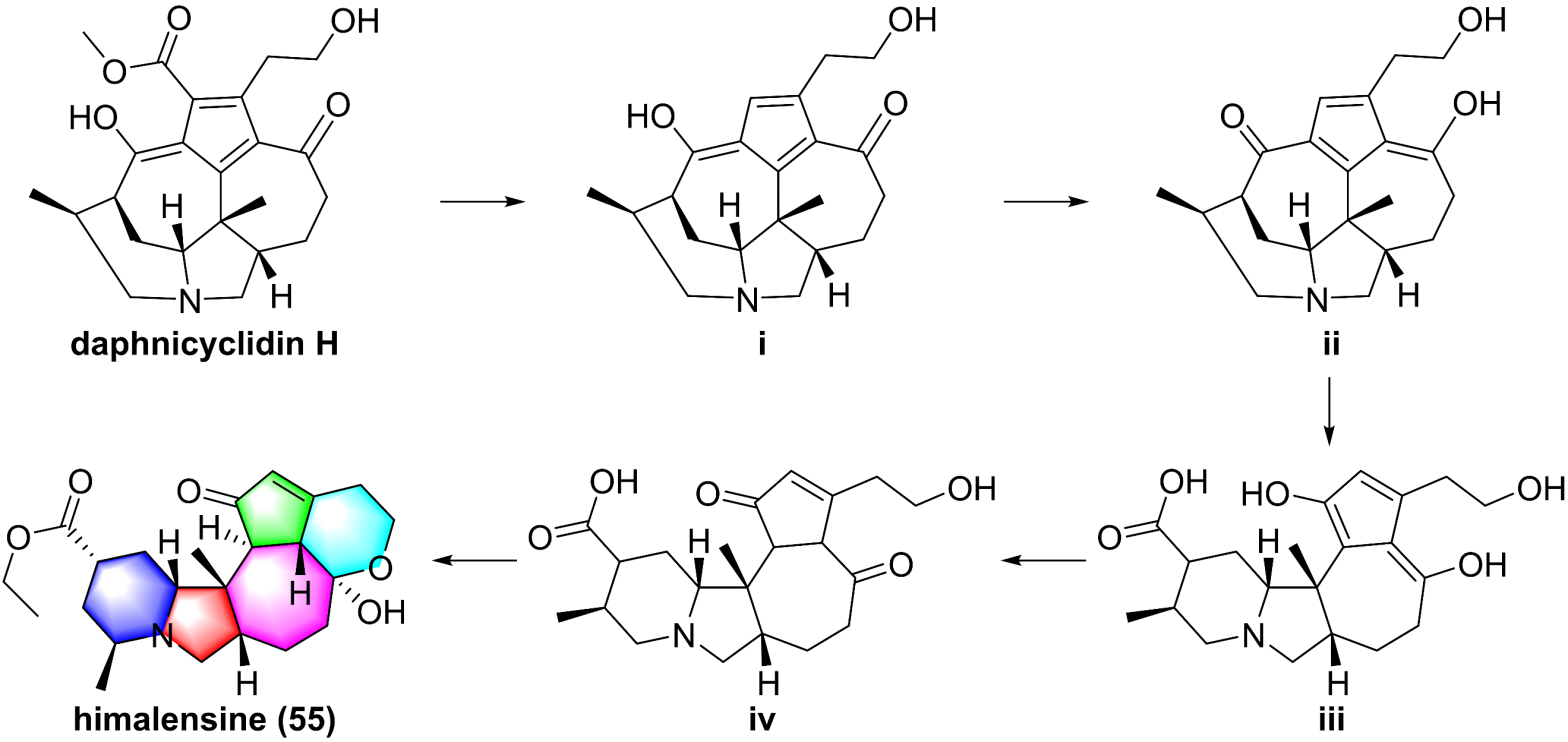
Proposed biosynthetic pathway of himalensine (**55**).

**Figure 15:**
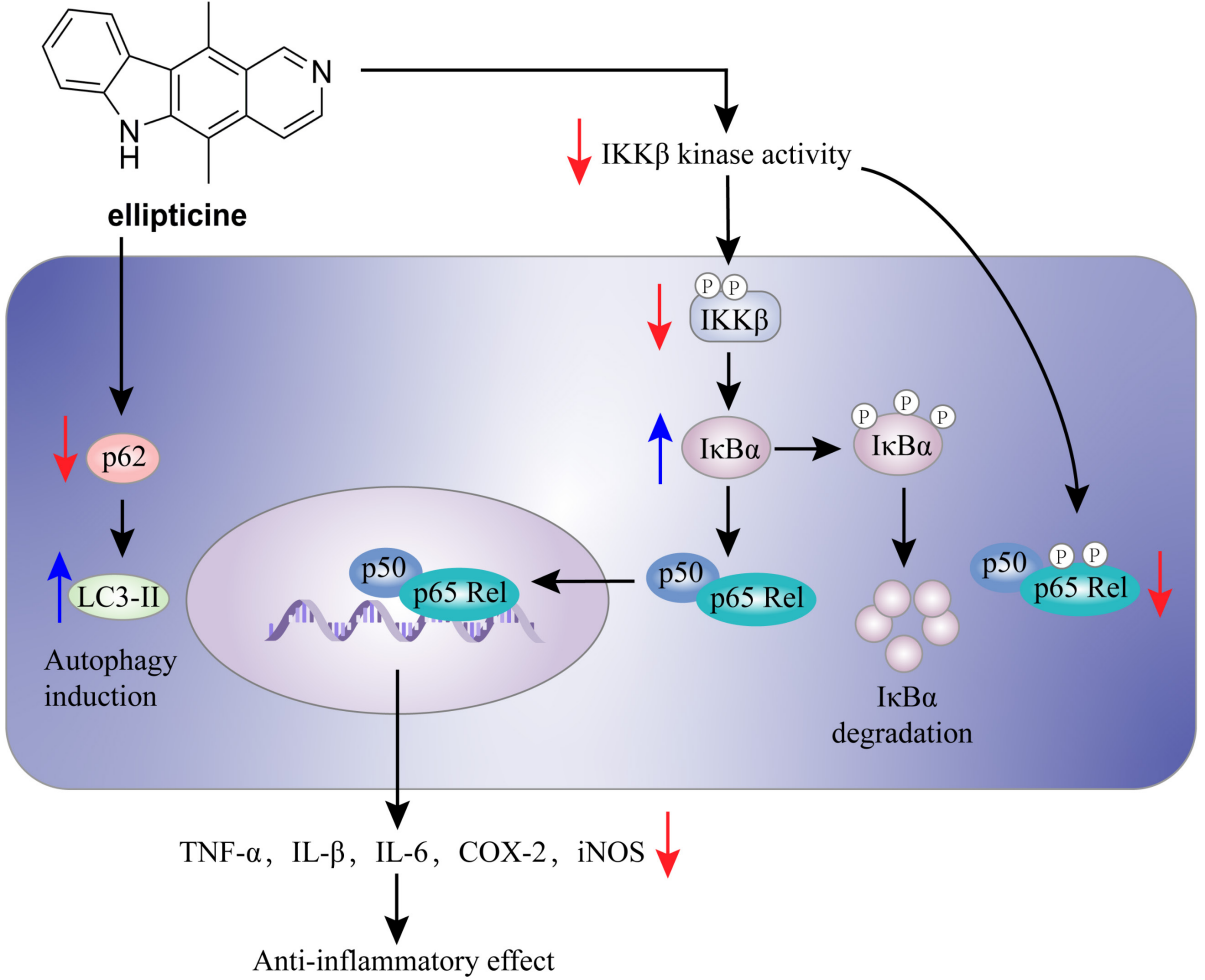
The mechanism of action of ellipticine (**56**) in the inflammation.

So far, fourteen natural alkaloids have been reported to possess inhibitory potentials towards IKKβ (**Figure 13-15**), including berberine (**43**), vinpocetine (**44**), cryptopleurin (**45**), matrine (**46**), tetrandrine (**47**), piperlongumine (**48**), daphnipaxianine A (**49**), pipernigamides E-G (**50-52**), tulipiferamide A (**53**), herbimycin A (**54**), himalesine (**55**), and ellipticine (**56**) 115, 161-169. Among them, himalensine (**55**, **Figure 14**), isolated from *Daphniphyllum himalense*, possesses an unprecedented 6/5/6/7/5/6 skeleton, and its proposed biosynthetic pathway was discussed 164. The decarboxylation of daphnicyclidin H, is a major constituent of *Daphniphyllum himalense*, yielded intermediate i, followed by Baeyer-Villager oxidation, ring opening, and esterification to afford **55** 164.

Herbimycin A (**54**) is an acetomycin antibiotic that possesses a potent Src tyrosine kinase inhibitory activity 170-172. Ogino and colleagues found that herbimycin A (**54**) could reduce the iNOS expression and chemokine production via inactivating NF-κB. Subsequently study demonstrated that herbimycin A (**54**) could bind to Cys59 in the KD region of IKKβ, resulting in the inhibition of the IKKβ activity, which was supported by the experiment of amino acid mutation (Cys59Ala) 173. A study by Chen et al. focused on the discovery of IKKβ inhibitors and their application in the inflammation demonstrated the potential of ellipticine (**56**, **Figure 15**), an alkaloid from *Ochrosia elliptica* 174*.* Ellipticine (**56**) could suppress the IKKβ activity through directly binding to IKKβ, resulting in the inactivation of NF-κB signaling pathway on the basis of kinase and binding experiments, and the result of amino acid mutation suggested that Cys46 in the activation loop of IKKβ played a role in the inhibition of ellipticine (**56**) 174*.*

### IKKβ inhibitors from chemical synthesis

#### Pyrimidines

**Figure 16:**
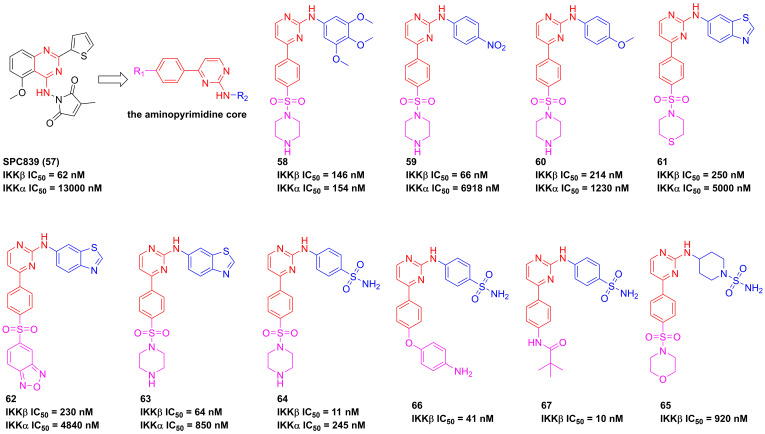
Aminopyrimidine type IKKβ inhibitors.

In 2001, Bhagwat et al. has reported a small molecule with an aminopyrimidine core, SPC839 (**57**, **Figure 16**), and its effect in an arthritis animal model 175. The pharmacological study on its action mechanism demonstrated its effect was based on the inhibition of SPC839 (**57**) on the IKKβ activity, which led to a new era of the development of IKKβ inhibitors. Bingham et al. summarized the structural characteristic of SPC839 (**57**) and built the aminopyrimidine scaffold to afford a series of aminopyrimidine analogues, such as **58**. Although this compound had a better potential than SPC839 (**57**), its selectivity was not satisfactory. Afterwards, they tried to modify the substituent moiety at the right of **58** through the induction of the electron-withdrawing (e.g. -CN, -NO_2_, and -Cl) or electron-donating (e.g. -OCH_3_ and -CH_3_) groups, and found that the electron-withdrawing group was in favor of the IKKβ selectivity (**59**, IKKα/IKKβ = 104.8; **60**, IKKα/IKKβ = 5.7; **61**, IKKα/IKKβ = 22.3) 175. The potential interaction of this type inhibitor with IKKβ was reported as well that Cys99 interacted with the backbone NH and the aminopyrimidine-nitrogen 176. Bingham and co-workers kept on the investigation of this type inhibitor, resulting in the production of **63** and **64** with an IKKβ selectivity 176. Subsequently, pharmaceutical chemists from Korea also synthesized piperidinyl aminopyrimidine inhibitors, such as **65**, whereas its potential had not been improved 177. Crombie' group from American and Hong' group from Korea re-designed the benzenesulfonamido aminopyrimidine scaffold through the introduction of other substituent groups at R_1_, respectively, and got two representative inhibitors **66** and **67** that possessed a low nanomole level of inhibitory potentials 178, 179. An analysis of their structures indicated that the sulfanilamide and amino groups formed three hydrogen bonds with Gly22, Asp103, and Lys106, respectively, except for the classical interactions of the aminopyrimidine backbone with Cys99. Additionally, **66** was applied for the investigation on LPS-mediated inflammation *in vitro*, and showed a remarkable anti-inflammatory effect 178.

#### Pyridines

**Figure 17:**
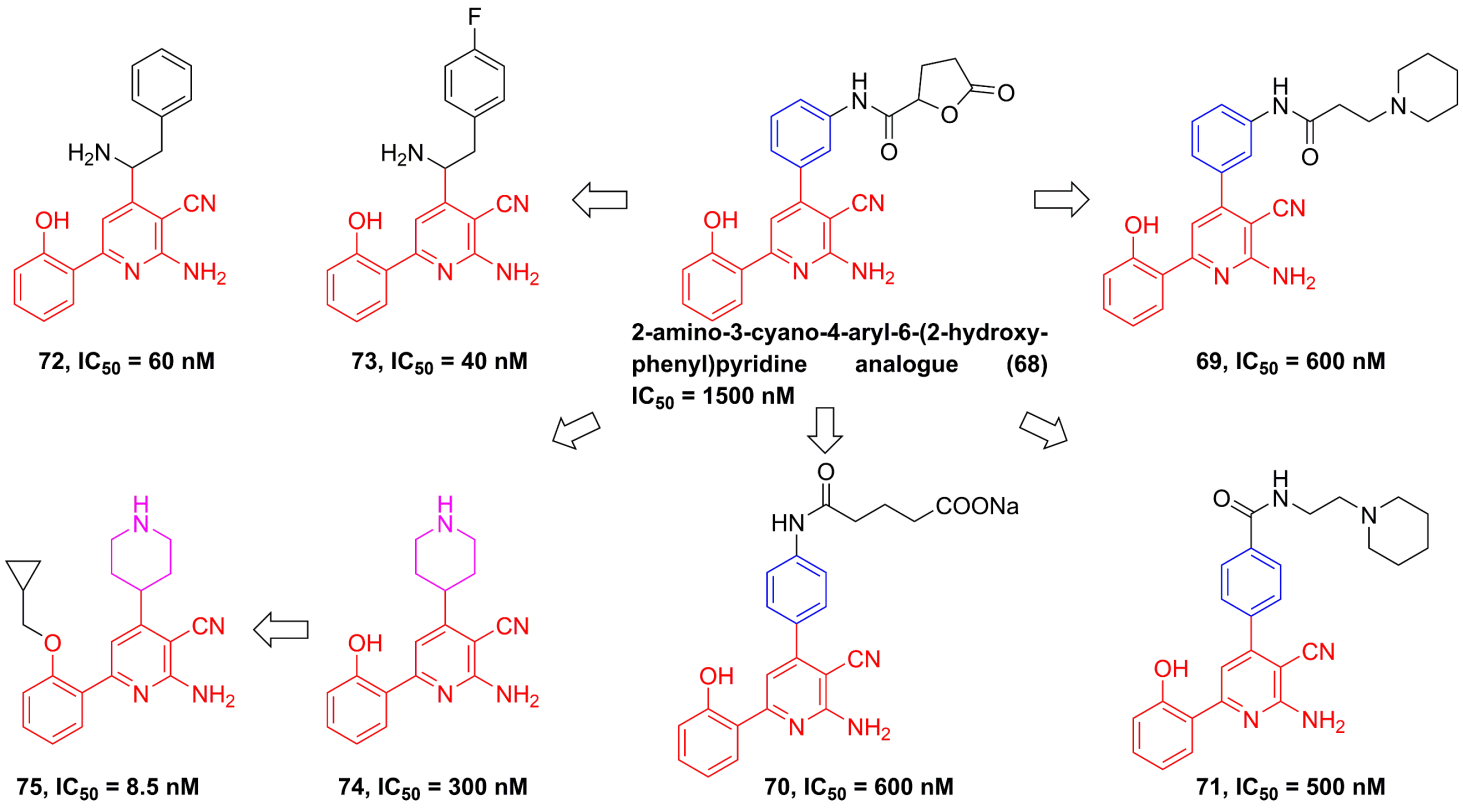
Optimization based on the skeleton of 2-amino-3-cyano-6-(2-hydroxy-phenyl)pyridine.

**Figure 18:**
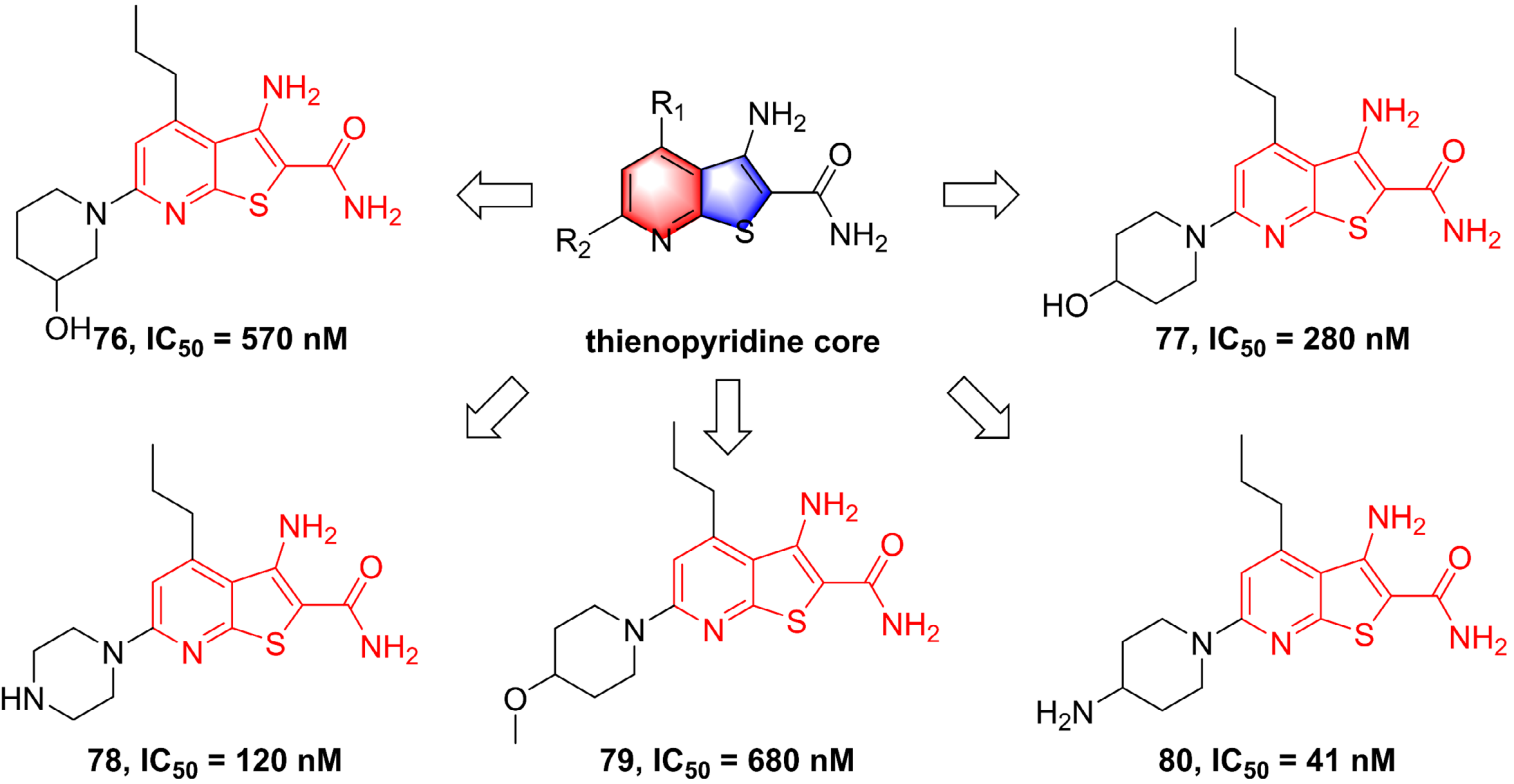
Optimization of thienopyridine core IKKβ inhibitors.

In 2002, Murata et al. performed the high-throughput screening based on the library of Bayer compounds, and found that 2-amino-3-cyano-4-aryl-6-(2-hydroxy-phenyl)pyridine analogue (**68**, **Figure 17**) displayed a potent inhibitory activity against IKKβ (IC_50_ = 1500 nM) 180. Based on the skeleton of 2-amino-3-cyano-6-(2-hydroxy-phenyl)pyridine, a series of inhibitors were designed and synthesized, yielding compounds **69-74** 180, 181. Among them, although compound **73** showed a low nanomole potential (IC_50_ = 40 nM), its performance was not as good as expected at cellular level (IC_50_ = 5000 nM) 181. After detailed analysis of their structure-activity relationship (SAR), a cyclopropyl analog **75** afforded from the structure of **74** not only displayed a low nanomole inhibitory effect (IC_50_ = 8.5 nM), but also possessed reasonable aqueous solubility (0.12 mg/mL in buffer), excellent permeability, and orally bioavailability 182. Based on previous studies, Wu et al. designed the thienopyridine skeleton (**Figure 18**) and optimized this type IKKβ inhibitor through the modification of R_1_ and R_2_ substituent groups 183. The SAR investigation indicated the role of n-propyl in R_1_ substituent group, and followed by the modification to produce selective inhibitors **76-80** 183. Meanwhile, **76-78** displayed anti-inflammatory effects against LPS-mediated TNF-α production *in vivo*, which provided a thought for the development of novel IKKβ inhibitors.

#### Pyrazines

**Figure 19:**
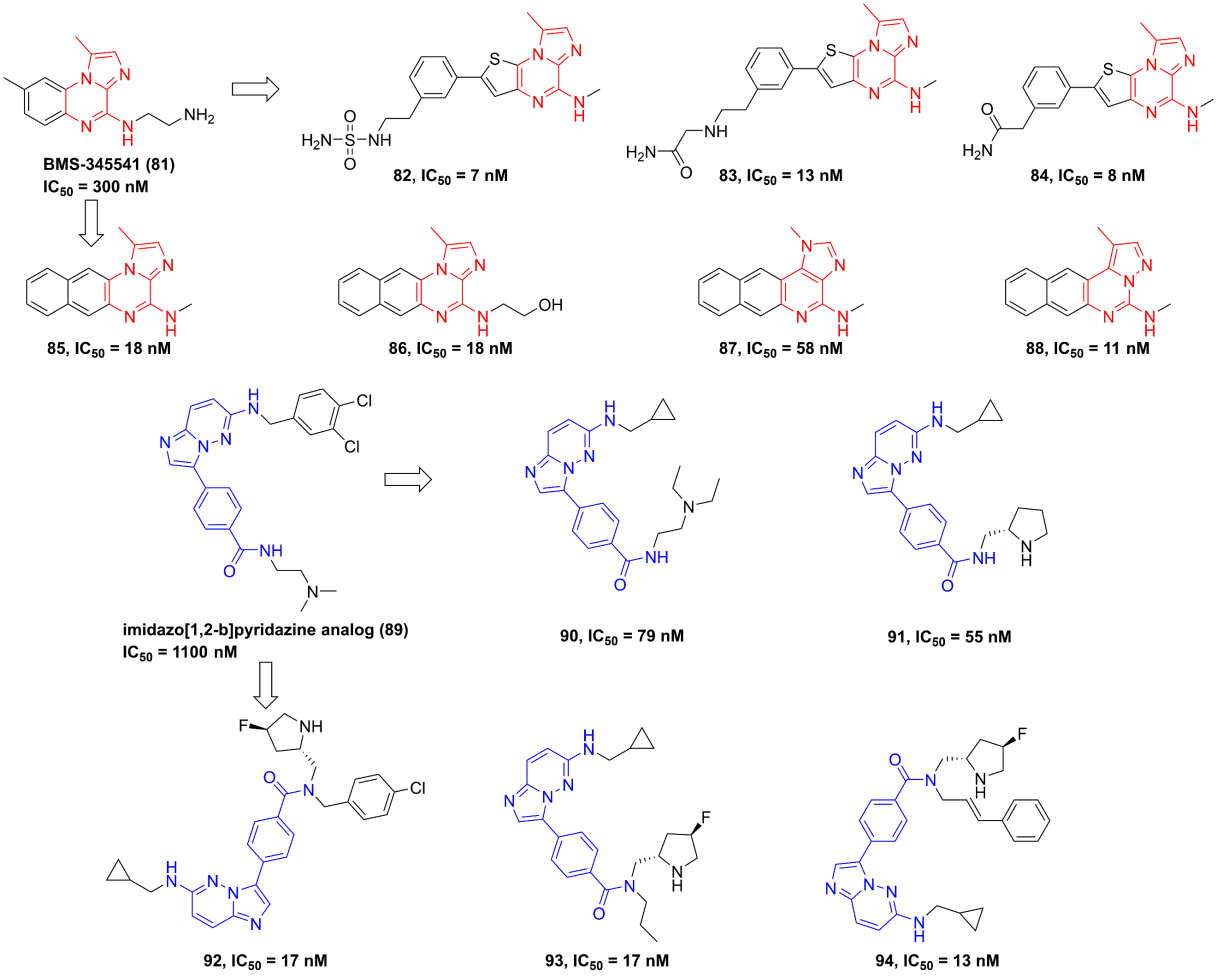
Pyrazine type IKKβ inhibitors.

In 2003, Burke and co-workers synthesized 4-(2′-aminoethyl)amino-1,8-dimethylimidazo(1,2-*a*)quinoxaline (BMS-345541, **81**, **Figure 19**) that was a selective IKKβ inhibitor (IKKα IC_50_/IKKβ IC_50_ = 13.3) *via* binding to the allosteric site of IKKβ 35. Furthermore, BMS-345541 (**81**) possessed an excellent pharmacokinetics and *in vitro* and *in vivo* anti-inflammatory effects through the release of LPS-mediated inflammatory factors 35, such as TNF-α, IL-1β, IL-8, or IL-6. According to the interaction with an allosteric site of IKKβ, a library of inhibitors with the imidazoquinoxaline core were developed and synthesized, such as **82-88** 184, 185. Among them, **83** not only possessed a low nanomole potential, but also displayed a good selectivity (IKKα IC_50_/IKKβ IC_50_ = 30). Meanwhile, the delightful pharmacokinetic property of **83** led to its perfect anti-inflammatory effect for reducing the release of LPS-mediated TNF-α *in vivo* 185.

Upon the high-throughput screening, Shimizu et al. also found the imidazo[1,2-*b*]pyridazine scaffold of **89**, and modified substituents in the 3-position of this type skeleton through the introduction of an electron-withdrawing group and hydrophobic substituents to the amide nitrogen to improve permeability and affinity for IKKβ. Therefore, they got many kinds of selective inhibitors **90-94** in 2010 and 2011, and summarized the interaction of this type inhibitor as well, requiring the role of Glu61 and Cys99 in the inhibition of IKKβ for pyrazine type IKKβ inhibitors 186, 187.

#### Quinoxalines and isoquinolines

**Figure 20:**
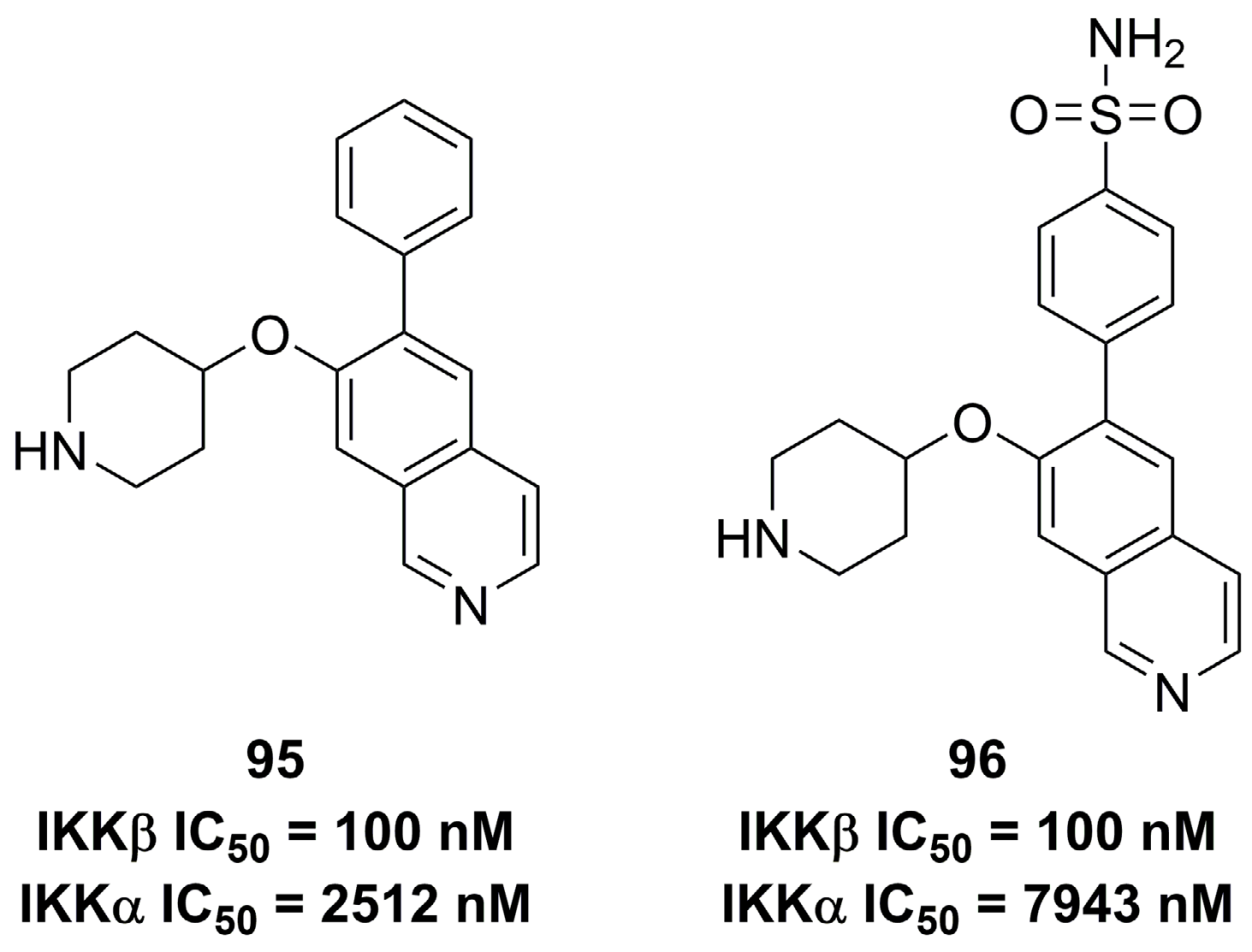
Isoquinoline type IKKβ inhibitors and its interaction with IKKβ.

Previously, Christopher's group disclosed 2-amino-3,5-diarylbenzamide inhibitors (**Figure 20**), and found the advantage of the sulfonamide moiety for the IKKβ inhibitory potency 188. Therefore, they applied a 3D pharmacophore on the basis of interactions between IKKβ and phenylcarboxamide inhibitors to filter the database down to 289 halides. Depended on this result, they designed and synthesized a series of potential inhibitors, such as **95** and **96**. These two compounds shared the same inhibitory potential against IKKβ and the special selectivity (IKKα IC_50_/IKKβ IC_50_, 25-fold for **95**; 79-fold for **96**), which was further supported by the molecular stimulation 188.

**Figure 21:**
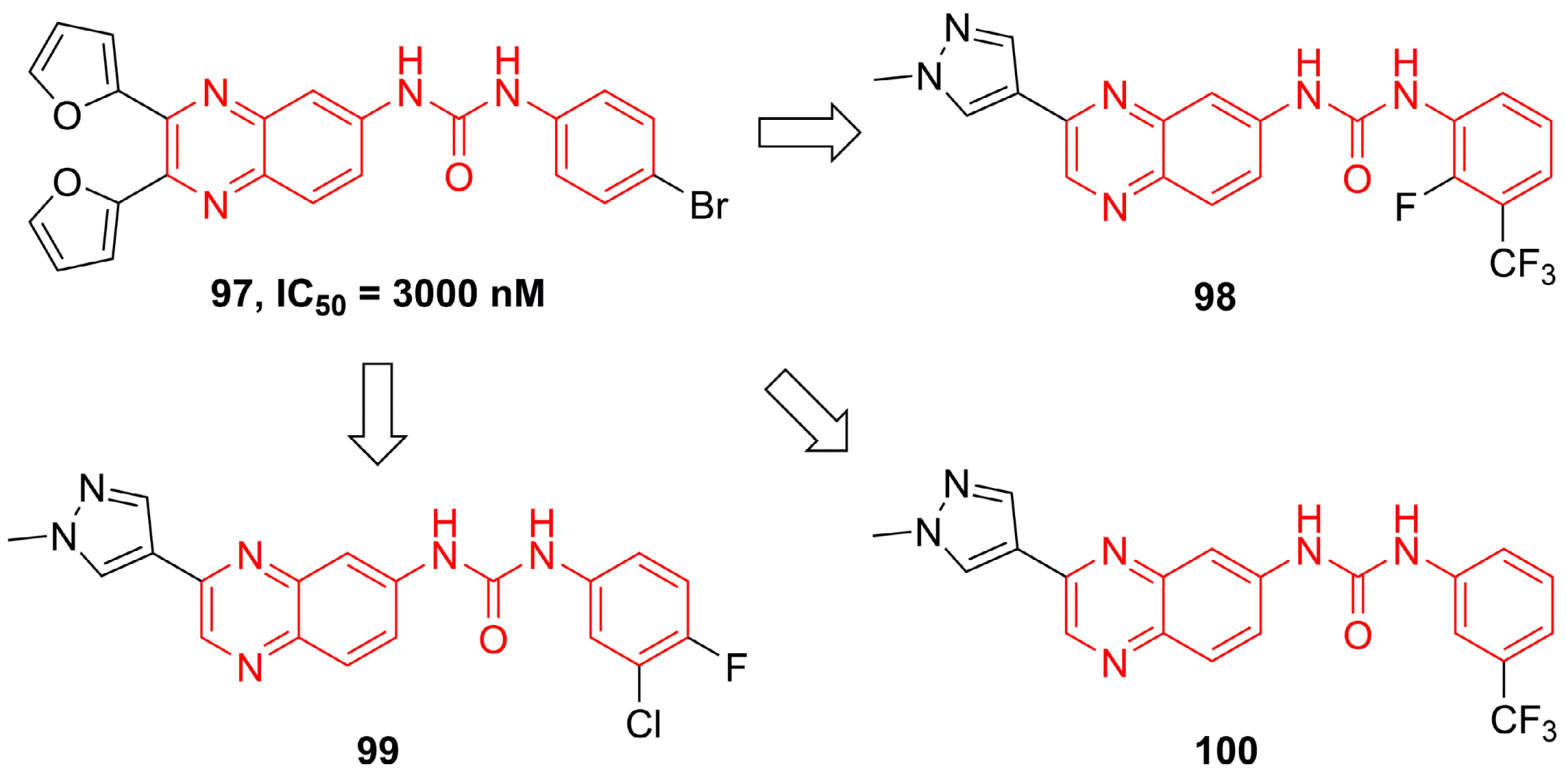
Quinoxaline type IKKβ inhibitors.

Recently, Radhakrishnan and co-workers found the hypothesis that IKKβ served as the signaling node to regulate the transcription and translation through the NF-kB and mTOR/p-S6K/p-eIF4EBP axis in tumors 189. Based on the result of the kinase screen experiment, they demonstrated the potential of a quinoxaline urea **97** (**Figure 21**) in the inhibition of the IKKβ activity, and used it to confirmed the hypothesis 189. In 2021, Radhakrishnan and colleges continued to optimize the structure of a quinoxaline urea inhibitor **97** and got the more potent inhibitors than the parent **97**, such as **98-100** 65. It was noteworthy that **98** not only showed the potent inhibitory effect but also possessed a good pharmacokinetics property. Preclinical results indicated the more potent anti-inflammatory (2.5 fold) and anti-tumor (4.3 fold) effects of **100** than the parent **97** 65, which provided a new direction for developing IKKβ inhibitors.

#### Benzamides

**Figure 22:**
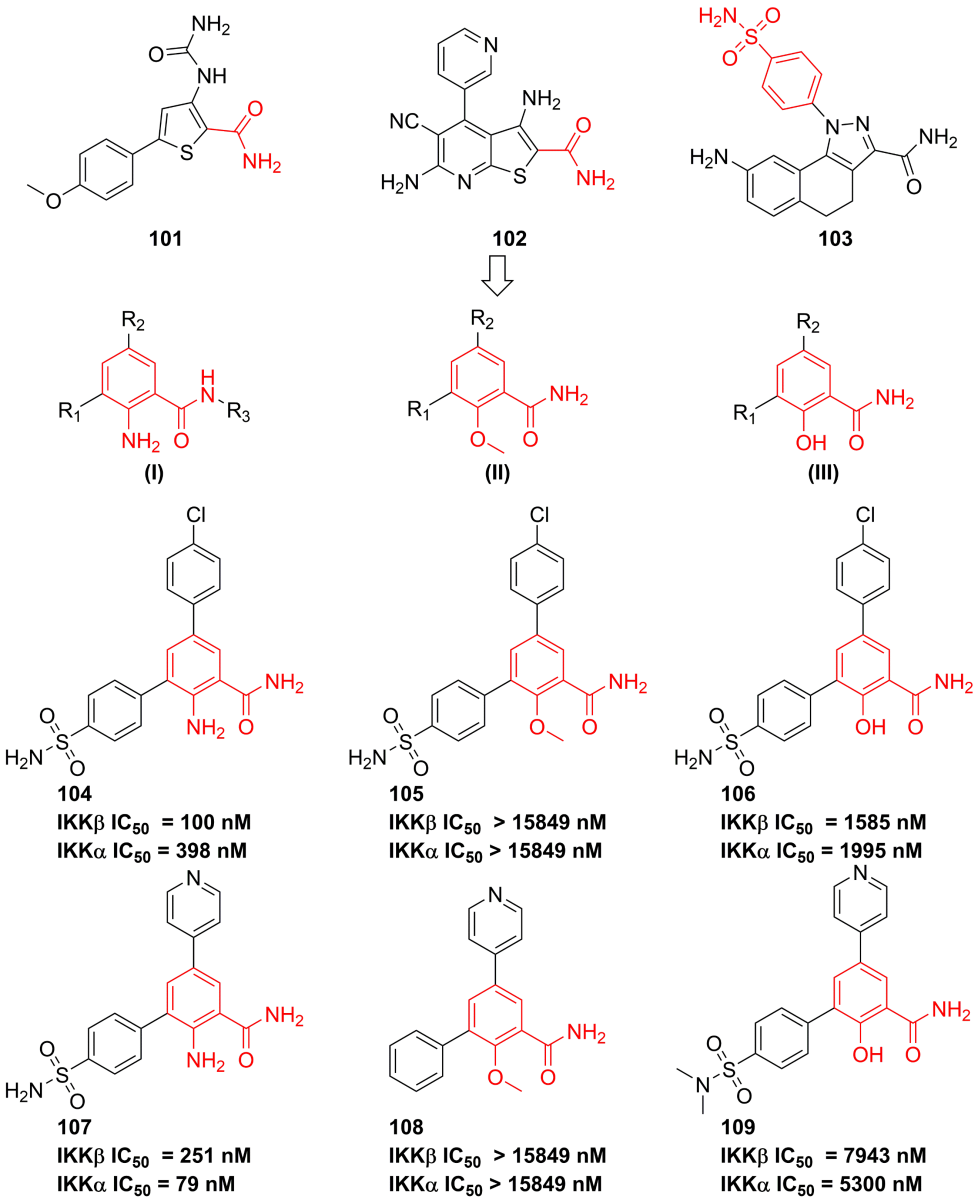
Optimization of benzamide type IKKβ inhibitors.

In 2007, Christopher and colleges analyzed recent IKK inhibitors, e.g. **101-103** (**Figure 22**), and found that **101-103** had a common motif, where the orientation of a primary amide was restricted by an adjacent hydrogen-bonding functionality, therefore, they tried to explore a new template based on the benzamide skeleton for developing IKKβ inhibitors 190. The 2-amino-benzamide scaffold by modifying the C-2 substituents was constructed by introducing the amino, hydroxy, or methoxy group to afford potent inhibitors, such as **104-109** 190. Although without the satisfactory result, this type skeleton displayed the potential as the temple of IKKβ inhibitors, which provided a valuable experience for developing IKKβ inhibitors.

#### Thiophenes and thiazolidines

**Figure 23:**
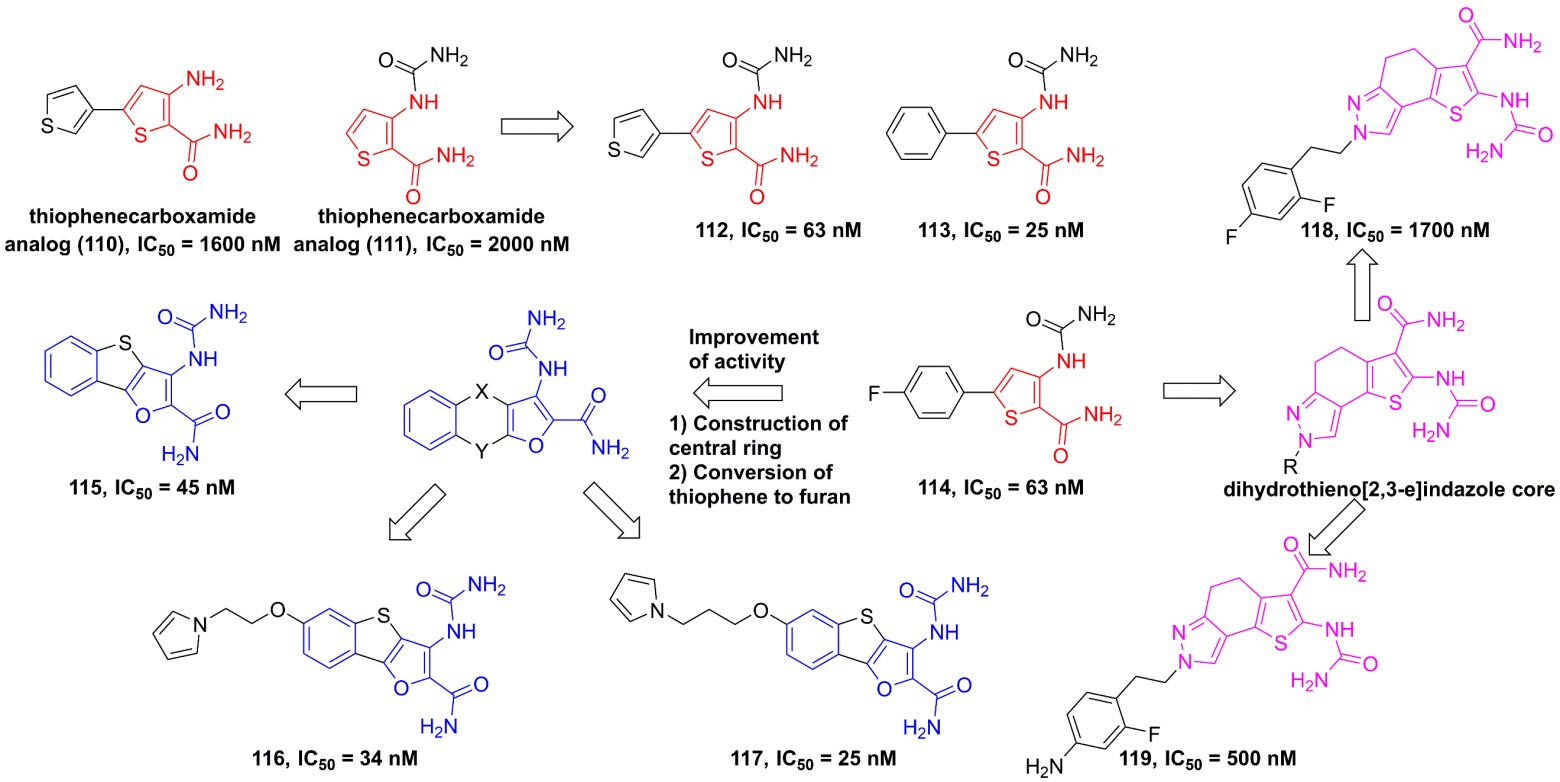
Optimization of thiophene type inhibitors.

In 2004, through the high throughput screening to find two potential thiophenecarboxamides **110** and **111** (**Figure 23**), Baxter et al. optimized the thiophene core to produce a series of analogs 191. Among them, compounds **112-114** showed low nanomole inhibitory activities against IKKβ and good pharmacokinetics 191. Analysis of the structural characteristic of thiophenecarboxamide **114**, a novel class of tricyclic furan derivatives were designed and synthesized by Sugiyama and colleagues, such as 3-[(aminocarbonyl)amino]-benzothieno-[3,2-*b*]furan-2-carboxamide derivatives **115-117** 192. Introduction of substituents onto the benzothieno[3,2-*b*]furan to overcome the low metabolic stability yielded a series of 6-alkoxy derivatives, meanwhile improved oral bioavailabilities of inhibitors. The potent inhibitory activity of these derivatives resulted from an intramolecular non-bonded S…O interaction, except for four hydrogen bond interactions with Glu97, Tyr98, and Cys99, which was further supported by the co-crystal result 192. A subsequent study by Takahashi et al. built a dihydrothieno[2,3-*e*]indazole core to afford a library of IKKβ inhibitors, such as **118** and **119**, while having unsatisfying inhibitory effects 193.

**Figure 24:**
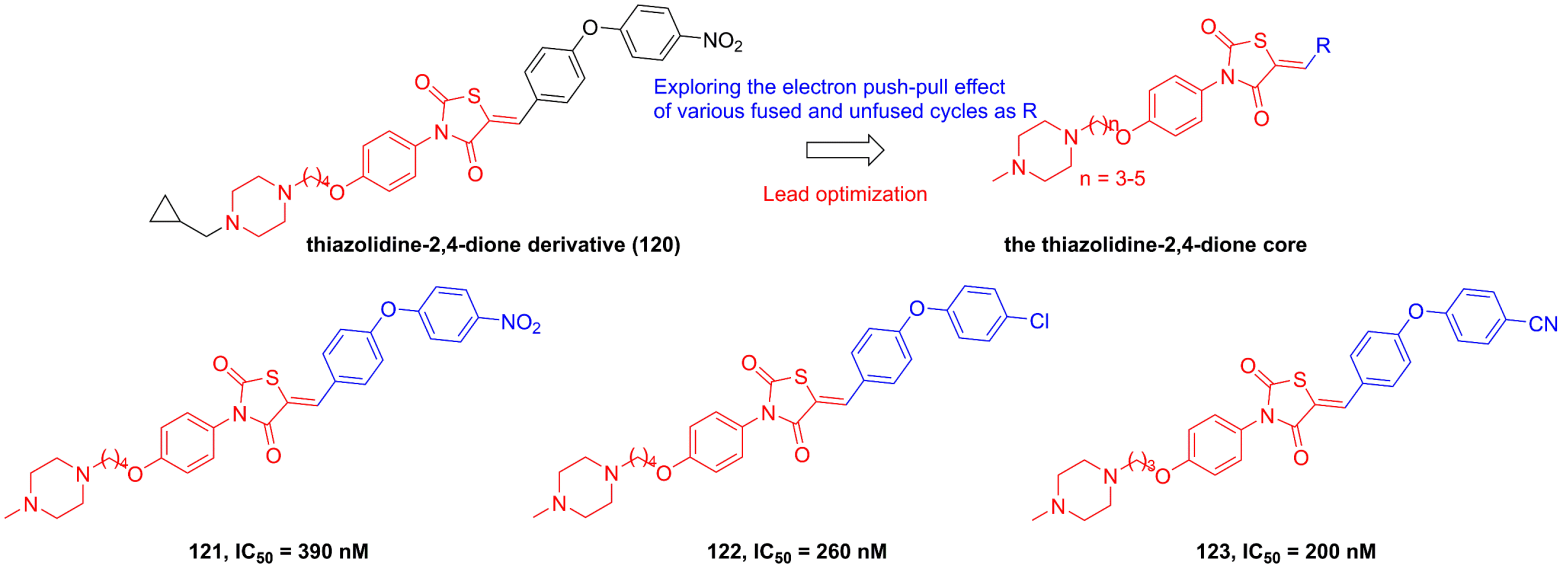
Optimization of thiazolidine IKKβ inhibitors based on the thiazolidine-2,4-dione core.

Roh's group found a lead compound (**120**,** Figure 24**) with a thiazolidine-2,4-dione skeleton and its potential in the inhibition of IKKβ (IC_50_ = 1500 nM), therefore, they tried to optimize this core via exploring the electron push-pull effect of the right moiety and analyze the SAR 194. Fortunately, **121** and **122** showcased more potent inhibitory effects than the parent (about 4-6 fold) 194. Subsequently, his group kept on the modification of the substituent depended on this skeleton, and got a library of the thiazolidine-2,4-dione derivatives. Among them, **123** with the cyano group at the right side possessed the sub nanomole level in the inhibition of IKKβ, meanwhile remaining the pharmacokinetics property and anti-inflammatory effect* in vitro*, which was further explained through the molecular stimulation showing the role of Arg47, Arg55, Trp58, and Leu91 for its potential 195. In the *in vivo* experiment, **123** enhanced the mortality of septic shock induced mice (80% survival), demonstrating its protective potential for septic shock 195.

#### Rhodanines

**Figure 25:**
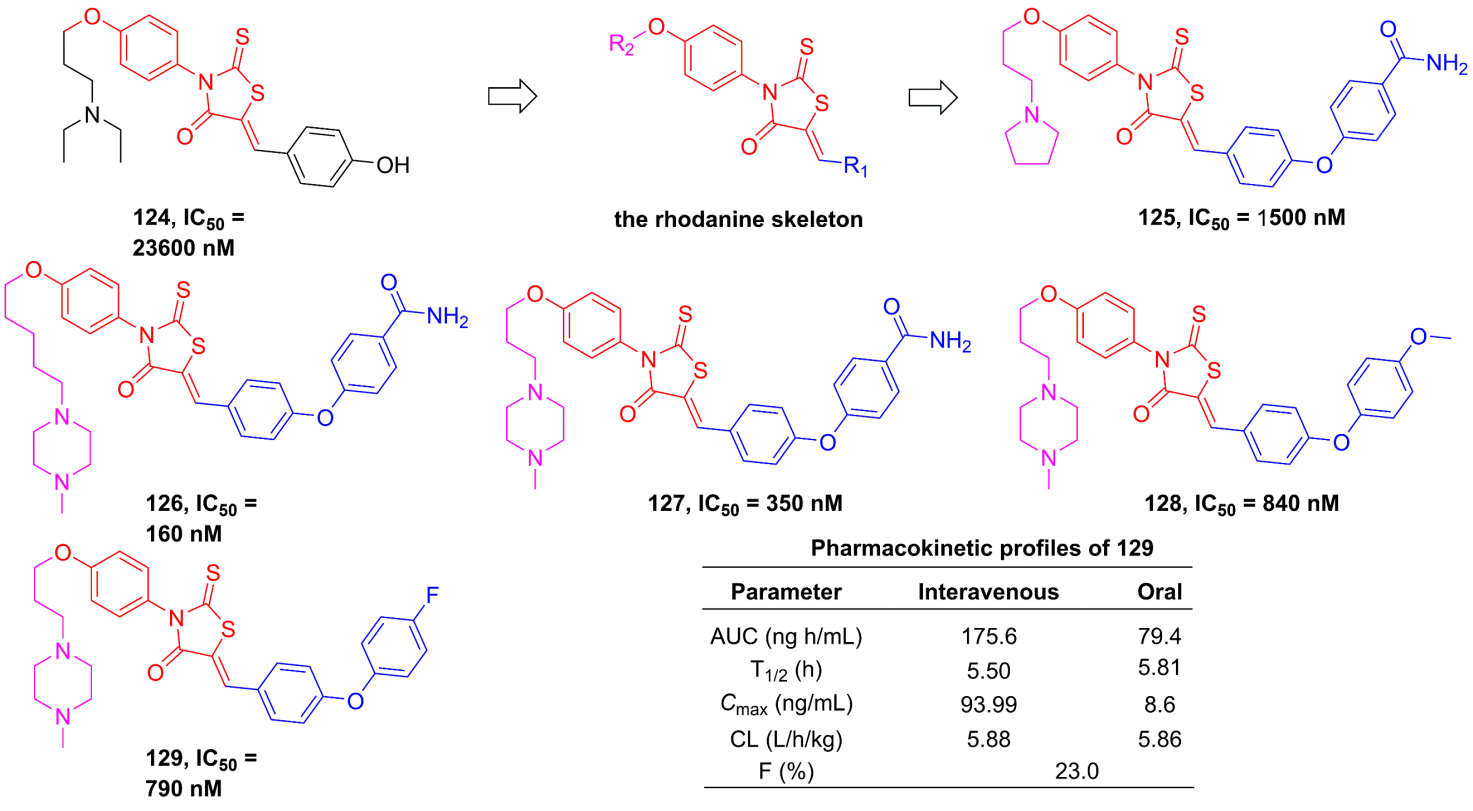
Rhodanine type inhibitors.

In 2012, Song et al. employed high-throughput screening to assay the inhibitory effect of the in-house library of compounds, in order to develop the new generation of IKKβ inhibitors 196. They found a hit compound **124** (**Figure 25**) sharing the rhodamine scaffold from druggable and transformative points of view 196. Therefore, the rhodanine core served as the basic skeleton to design IKKβ inhibitors, such as **125-127**, and the result of the kinase assessment indicated the selectivity of **127** in the inhibition of the IKKβ inhibitors as well 196. Although the good selectivity and inhibitory effect of this type compound, **125-127** did not display satisfactory pharmacokinetic property. Skin's group subsequently optimized its metabolic property through the modification of the right moiety based on the structure of **127**, resulting in the production of** 128** and **129** 197. It was very excited that **129** remained a selectivity and inhibitory effect of the rhodanine type inhibitors, while possessed a good metabolic stability (T_1/2_ = 239 min in microsome; T_1/2_ = 89 min in plasma) and pharmacokinetic character 197.

## Conclusion and perspective

In summary, we described the structure and transduction mechanism of IKKβ and its inhibitors from natural products (e.g. sesquiterpenoids, diterpenoids, flavonoids, and alkaloids) and chemically synthesized (e.g. pyrimidines, pyridines, pyrazines, and benzamides). Furthermore, inhibitory potentials of IKKβ are associated with in various diseases through regulating the NF-κB pathway, including cancer, PD, diabetes, SCI, NAFLD, osteolysis, and arthritis, therefore, it has been considered a potential target. Notably, for inhibitors from chemically synthesized, pyrimidine- and pyrazine-type compounds display low nanomole inhibitory effects, which attributes to the aromatic nitrogen atom in the skeleton of pyrimidine and pyrazine due to its hydrogen bond interaction with Cys99 of IKKβ, but their physical properties (water solubility), bioavailability, and pharmacokinetic parameters remains great improving spaces. Although the clinical trial of IKKβ inhibitors, such as IMD-2560, and SAR-113945, do not show satisfactory results for the safety, efficacy, and selectivity, natural products with more complex and changeable structural skeletons offer endless possibilities for discovering IKKβ inhibitors. Among naturally occurred inhibitors, sesquiterpenoids with an α,β-unsaturated lactone moiety have great potentials because they covalently bind to amino acid residues Cys46 and Cys179 of IKKβ. However, the total synthesis of natural products with multiply chiral centers has always been a difficult problem, therefore, simplification and extraction of effective structures of natural inhibitors may be an efficient means to synthetize new type IKKβ inhibitors in the future.

## Abbreviations

Aβ: amyloid beta
AD: Alzheimer's disease
CAC: colitis-associated cancer
CNS: central nervous system
COX-2: cyclooxygenase-2
DNA: deoxyribonucleic acid
HFD: high-fat diet
IgG: immunoglobulin G
IKK: inhibitory kappa B kinase
IKKβ: inhibitory kappa B kinase beta
IκB: inhibitors of kappa B
IL-6: interleukin-6
iNOS: inducible nitric oxide synthase
KD C: C-terminal kinase domain
KD N: N-terminal kinase domain
KO: knockout
LPS: lipopolysaccharide
MEKK3: mitogen-activated protein kinase kinase kinase 3
MPTP: 1-methyl-4-phenyl-1,2,3,6-tetrahydropyridine
NAFLD: non-alcoholic fatty liver disease
NAK: NF-κB activating kinase
NASH: non-alcoholic steatohepatitis
NF-κB: nuclear factor kappa-B
NIK: NF-κB-inducing kinase
NLS: nuclear localization sequence
PD: Parkinson's disease
RANKL: receptor activator of nuclear factor-κB ligand
SCI: spinal cord injury
SDD: scaffold dimerization domain
SNP: single nucleotide polymorphism
TAK1: TGF-beta-activated kinase 1
TNF-α: tumor necrosis factor alpha
TRAF6: TNF receptor-associated factor 6
ULD: ubiquitin-like domain
